# Deep Reinforcement Learning-Based Self-Optimization of Flow Chemistry

**DOI:** 10.1021/acsengineeringau.5c00004

**Published:** 2025-05-13

**Authors:** Ashish Yewale, Yihui Yang, Neda Nazemifard, Charles D. Papageorgiou, Chris D. Rielly, Brahim Benyahia

**Affiliations:** † Department of Chemical Engineering, 5156 Loughborough University, Loughborough, Leicestershire LE11 3TU, U.K.; ‡ Synthetic Molecule Process Development, Process Engineering and Technology, Takeda Pharmaceuticals International Company, 40 Landsdowne Street, Cambridge, Massachusetts 02139, United States

**Keywords:** flow chemistry, self-optimization, deep reinforcement learning, deep deterministic policy gradient, adaptive hyperparameter tuning, Bayesian optimization

## Abstract

The development of effective synthetic pathways is critical in many industrial sectors. The growing adoption of flow chemistry has opened new opportunities for more cost-effective and environmentally friendly manufacturing technologies. However, the development of effective flow chemistry processes is still hampered by labor- and experiment-intensive methodologies and poor or suboptimal performance. In this context, integrating advanced machine learning strategies into chemical process optimization can significantly reduce experimental burdens and enhance overall efficiency. This paper demonstrates the capabilities of deep reinforcement learning (DRL) as an effective self-optimization strategy for imine synthesis in flow, a key building block in many compounds such as pharmaceuticals and heterocyclic products. A deep deterministic policy gradient (DDPG) agent was designed to iteratively interact with the environment, the flow reactor, and learn how to deliver optimal operating conditions. A mathematical model of the reactor was developed based on new experimental data to train the agent and evaluate alternative self-optimization strategies. To optimize the DDPG agent’s training performance, different hyperparameter tuning methods were investigated and compared, including trial-and-error and Bayesian optimization. Most importantly, a novel adaptive dynamic hyperparameter tuning was implemented to further enhance the training performance and optimization outcome of the agent. The performance of the proposed DRL strategy was compared against state-of-the-art gradient-free methods, namely SnobFit and Nelder–Mead. Finally, the outcomes of the different self-optimization strategies were tested experimentally. It was shown that the proposed DDPG agent has superior performance compared to its self-optimization counterparts. It offered better tracking of the global solution and reduced the number of required experiments by approximately 50 and 75% compared to Nelder–Mead and SnobFit, respectively. These findings hold significant promise for the chemical engineering community, offering a robust, efficient, and sustainable approach to optimizing flow chemistry processes and paving the way for broader integration of data-driven methods in process design and operation.

## Graphical abstract

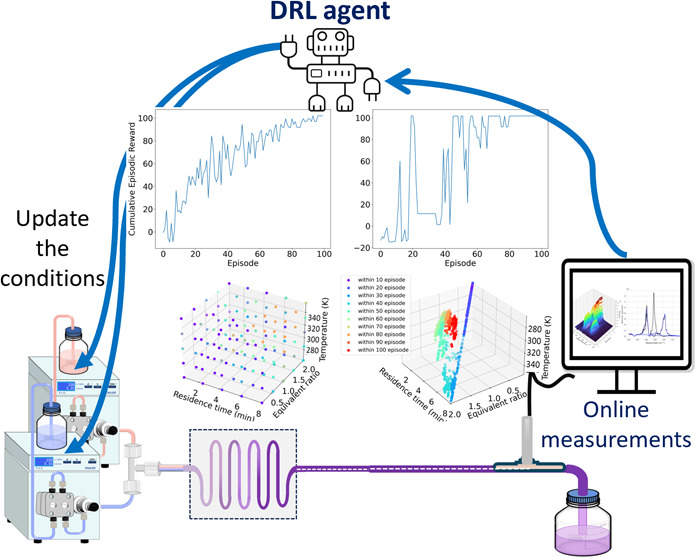

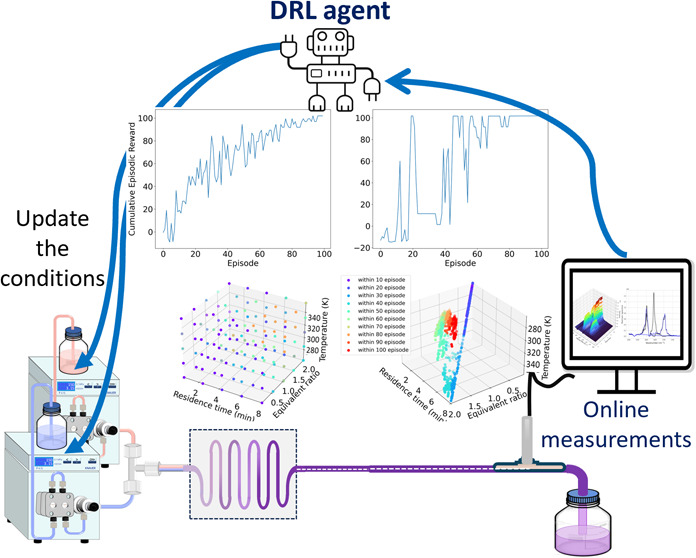

## 1 Introduction

Chemical reactions are essential steps to synthesis a variety of products, intermediates, additives, etc., relevant to several key industries such as the pharmaceutical, agrochemical, and petrochemical sectors. The total production costs are largely determined by the performance of the synthetic steps and inherent purification technologies, besides product quality and safety, environmental performance, and overall economic viability. The performance of a reaction is commonly captured by yield and selectivity which are influenced by a complex interplay between various factors such as catalysts, solvents, substrate concentrations, temperature, and reaction technologies which must be optimized to deliver consistent product quality. Traditional optimization approaches are labor-intensive, time-consuming, and very costly, often relying on “one variable at a time” (OVAT) experimentation or human intuition. The development of effective synthetic pathways for active pharmaceutical ingredients in pharma is a painstaking and very costly process.^1,2^ Poor reaction pathways may impact not only costs due to poor yield but also safety of the drug. The presence of impurities or side products makes the purification and isolation steps more challenging^3,4^ and may in many cases jeopardize the clinical trials and, if not addressed, may result in product withdrawals, leading to significant financial and reputational damage.^5–7^

To address these challenges, it is important to design more effective synthetic strategies and technologies and develop smarter and more systematic optimization strategies to avoid pitfalls and limitations of the traditional optimization methods and human intuitions. Continuous manufacturing and flow chemistry are increasingly adopted in the pharmaceutical industry to deliver more cost-effective, eco-friendly, and resilient alternatives to the traditional batch processing.^8,9^ Most importantly, the interplay between flow chemistry, advanced real-time process analytical technologies, and self-optimization can open a new era for effective development, design, and operation based on plug-and-play strategies. To deliver these objectives, it is critical to develop the next generation algorithms and methodologies specifically to address this new class of real-time optimization problems. Moreover, Industry 5.0, leveraging advancements in artificial intelligence, big data analytics, and autonomous robotics, is emerging as a transformative approach to enhancing efficiency and minimizing risks in chemical reactions.^10–13^ Recently, several machine learning (ML) methods have been successfully implemented to analyze more effectively both historical and real-time experimental data, delivering unique insights and allowing more reliable decision-making and effective design and operation strategies.^14,15^

Additionally, high-throughput experimental platforms for automated reaction optimization are increasingly adopted to maximize experimental screening.^16,17^ These methods and technologies may offer enhanced and systematic exploration of the reaction recipes and conditions (e.g., reagents, solvents, catalysts). However, they are often restricted to batch processes and can be extremely costly due to, due their intensive experimentation nature, particularly when implemented based on trial-and-error, instead of rigorous optimization strategies. The combination of flow chemistry and advanced optimization and control algorithms is opening new avenues for more effective real-time monitoring and adjustment of the reaction conditions. Over the past few years, researchers have implemented several traditional optimization methods such as Nelder–Mead simplex algorithm,^18,19^ stable noisy optimization by branch and fit (SnobFit) algorithm^20–22^ and mixed integer nonlinear programming (MINLP).^23^ Additionally, Deep Neutral Networks (DNNs) were used to optimize residence time, temperature, and catalyst loading in the Pd-catalyzed Suzuki-Miyaura reaction, predicting yield and identifying optimal reaction conditions by exploring the available reaction space.^24^ However, these approaches can be data extensive and may require large sets of expensive experiments.^25^ Among several other ML methods, Bayesian optimization has gained increased popularity.^26–28^ The method uses kernel density estimators to explore the decision variable space and, by balancing exploitation and exploration, optimally identifies the next experimentation/sampling locations. Shields et al.^28^ implemented experimental design based on Bayesian optimization featuring several strategies, such as density functional theory, cheminformatic, and binary one-hot encoded, aimed to find the optimal reaction conditions and functional groups of reactants. The authors proposed a new encoding of the reactions by concatenating molecular descriptors for each chemical component and continuous variables (temperature, reaction time, and concentration). Schweidtmann et al.^29^ developed a new package, Thomson Sampling Efficient Multi-Objective (TS-EMO), to support initial sampling methods and Bayesian optimization with Gaussian processing surrogate models. The tool was used to find optimal reaction conditions for two different organic reactions, maximizing space-time yield and minimizing the E-factor. The applicability of Bayesian optimization is extended to different areas such as multistep reactions,^30,31^ multiobjective problems,^32,33^ and multitask Bayesian optimizations.^34,35^ Furthermore, Bayesian optimization supported with Gryffin can operate with both continuous and discrete variables.^36,37^ Overall, data-driven approaches may help minimize human bias and accelerate the optimization and development processes, enhancing precision and efficiency in synthetic route development. Despite these advantages, ML models, in particular, may face challenges when addressing new or unknown conditions due to their reliance on labeled data and pattern recognition techniques. These models also require substantial amounts of data for effective training, and interpreting their results can be complex. Alternatively, reaction optimization can be effectively addressed using reinforcement learning (RL). As a subset of machine learning, RL focuses on optimizing action policies to maximize or minimize rewards based on continuous interactions with the environment. Deep reinforcement learning (DRL), which employs DNNs to estimate unknown functions of state and action spaces (such as policy or value functions), has proven powerful in many applications. By deriving estimations from a small sample of observations, DRL addresses the challenges of high-dimensional state spaces and solves increasingly complex problems.^38^ DRL’s impactful applications span numerous fields such as vision-based navigation and manipulation in robotics,^39,40^ autonomous navigation in self-driving cars,^41,42^ and optimization of energy distribution in smart grids and buildings.^43–45^ DRL also revealed pivotal in the control of crystallization processes for pharmaceuticals,^46,47^ and optimization of the production lines in the manufacturing industries.^48,49^ Most importantly, DRL has shown capabilities to effectively manage complex chemical processes by dynamically adjusting conditions in real-time. For instance, DRL was effectively applied to reaction control in polymerization processes by integrating a Long Short-Term Memory (LSTM) network to represent surrogate modeling and proximal policy optimization for decision making.^50,51^ In addition, Zhou and coauthors^52^ used a recurrent neural network as a policy function for decision making to optimize chemical reactions. Due to the time-consuming nature of evaluating these reactions, their approach involved initially training the model on simulated reaction data using a mixture of Gaussian density functions. This probabilistic approach models a data set as a weighted combination of multiple Gaussian distributions, each characterized by its own mean and covariance. It enhanced performance was achieved by continuously approximating the response surface of the chemical reaction systems, capturing their complex behavior with more reliable models. This pretraining strategy was designed to approximate complex reaction decision spaces and effectively handle multiple local minima. To improve exploration and avoid local optima traps, the authors introduced a randomized exploration strategy. This strategy uses a stochastic recurrent neural network to generate random decision choices, enhancing the algorithm’s ability to explore different experimental conditions more effectively. Their method achieved optimal reaction conditions in just 40 episodes, delivering a significant improvement over the traditional methods such as covariance matrix adaptation, evolution strategy, and OVAT approaches. More recently, Neumann and Palkovits^53^ demonstrated the capability of DRL in optimizing methane oxidation in a plug flow reactor, highlighting the potential of self-optimization in flow chemistry. However, their approach relies on fixed hyperparameters and predefined target conditions, which can be particularly limiting in chemical reaction optimization. In these systems, even slight variations in reaction parameters can significantly alter outcomes, and a rigid framework may fail to explore the full parameter space.^54^ Additionally, as seen in continuous control tasks,^55^ the sample inefficiency and high computational cost associated with simulating numerous reaction iterations pose substantial challenges. Moreover, analogous issues have been observed in related fields such as de novo drug design^56^ and in automated chemical synthesis,^57^ where balancing exploration with computational expense is critical. Future work could benefit from integrating adaptive hyperparameter tuning and more robust exploration strategies to better capture the dynamic behavior of chemical systems, thereby improving the discovery of true optimal reaction conditions.

Addressing these limitations could enhance DRL’s application in reaction optimization, including incorporating more complex scenarios and benchmarking against traditional methods.

In this paper, we propose addressing the challenges discussed earlier by introducing DRL, allowing more effective optimization of the reaction conditions of continuous chemical reactions. DRL combines the strengths of deep learning and reinforcement learning, allowing the development of strategies that can handle high-dimensional state and action spaces, which is critical in the complex environment of continuous action spaces. Deep Deterministic Policy Gradient (DDPG), a model-free, off-policy actor-critic method, is well-suited for such environments, as it allows agents to navigate an infinite number of possible actions efficiently.^58,59^ The model-free nature of DDPG facilitates the self-optimization of reaction conditions by enabling autonomous decision-making, where RL agents select actions that maximize expected rewards without human inputs. The proposed DDPG agent uses deep learning capabilities to extract meaningful features from complex data and consolidates it by the reinforcement learning power to learn optimal policies through interaction with the environment. It simplifies the learning process and enhances sample efficiency by focusing on learning a deterministic policy that directly maps states to actions, rather than learning a distribution over actions (stochastic approach) that requires more exploration and data. The performance of the RL agent is further enhanced by optimizing hyperparameters and set up of the episodes and actions. The proposed DRL self-optimization strategy is validated using a case study relevant to flow chemistry, offering a continuous, adaptive, and scalable approach that enhances efficiency, resource utilization, and cost-effectiveness. The imine synthesis, which exhibits side reactions, is used to show the capabilities and performance of the proposed approach.

Additionally, the DDPG agent’s performance is compared against the state-of-the-art gradient free optimization techniques, highlighting the effectiveness of DDPG in achieving superior optimization outcomes.

## 2 Method

The proposed general methodology to address the self-optimization problem is outlined in the workflow presented in Figure 1. The process begins with the definition of the environment, including decision variables, states, and outputs, which sets the foundation for the subsequent steps. An RL agent is then designed to interact with the environment, followed by the formulation of a reward function to steer the agent toward achieving the desired behaviors. The agent undergoes a training phase involving the hyperparameter optimization to identify and fine-tune key hyperparameters. After training, the agent is tested to evaluate its performance and robustness. Finally, the experiment is conducted under optimal conditions to validate the effectiveness of the trained agent.

**Figure 1:**
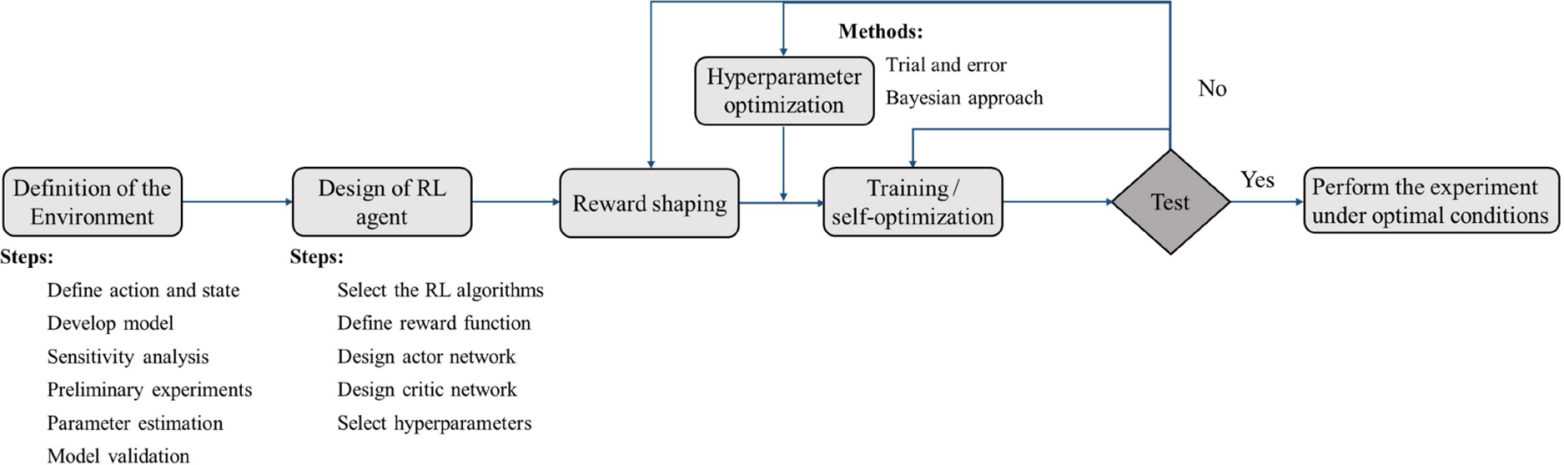
Workflow of the proposed deep reinforcement learning-based self-optimization method.

### 2.1 Environment

The environment represents the system of interest which can be influenced by a set of manipulated or decision variables which are captured by the set of actions initiated by the agent at a given time. The response of the system to a set of actions is captured by the states and outputs which allow the evaluation of instantaneous and cumulative rewards. In this study, the environment is represented by a mathematical model which allows more cost-effective training before validation or deployment in the real system. The imine synthesis in a tubular reactor (continuous) is considered as a case study to validate the proposed approach. The Imines and their derivatives are considered as key intermediates for the synthesis of nitrogen heterocycles and versatile pharmacophores.^60^ In addition, they are used in several important classes of compounds with herbicidal, anticancer, and antimycotic activities.^61^ The imine synthesis considered in this study is a condensation reaction between benzylamine **1** (ReagentPlus, ≥99%, Sigma-Aldrich, United Kingdom) and benzaldehyde **2** (ReagentPlus, 99%, Sigma-Aldrich, United Kingdom) in the presence of methanol (for synthesis, Fisher Scientific, United Kingdom), to produce imine *N*-benzylidenebenzylamine **3** as shown in Scheme 1.

**Scheme 1:**
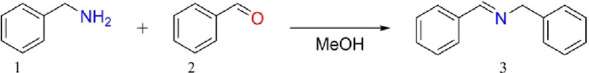
Imine Synthesis^a^

a Benzaldehyde **1**, benzylamine·**2**, and *N*-benzylidenebenzylamine **3**.

#### 2.1.1 Mathematical Model

The mathematical model of the imine synthesis system in flow is used as the environment for the RL-based self-optimization. The mathematical model was constructed using the following key assumptions:

- The reaction is homogeneous.
- No axial dispersion (plug flow reactor).
- The feeding temperatures (*T*_f_) and initial reactor temperature (*T*_0_) are equal.
- The overall heat transfer coefficient is constant over the length of reactor and during the reaction.
- The density of the reaction mixture (ρ) is constant.
- Chemical reactions are the only processes that can produce heat, and the cooling system is the only way for heat removal (no heat loss).

The residence time (Res_*t*_) of the flow reactor can be expressed as a function of the total volumetric flow rate (*Q*) and the reactor volume (*V*) by eq 1:

$${\mathrm{Res}}_{t}=\frac{V}{Q}$$ 1

The concentration of each reactant is determined by its individual flow rate relative to the total feed rate (*Q*) and concentration of the stock solution of the reactant. The total flow rate (*Q*) is the sum of the individual flow rates of all reactants.

The concentration of each reactant (*c*_*j*f_) in the feed stream is described as

$$c_{jf}=\frac{Q_{j}}{Q}\times C_{sj}$$ 2

where *Q*_*j*_ is the individual flow rate and *C*_s*j*_ is the concentration of the stock solution of the reactant, *j*, respectively.

The mass balance for the reactants and product in the tubular reactor are shown in the partial differential equation below, which depicts the spatial-temporal changes in the concentration of the different species.

$$\frac{{\partial c}_{i}}{\partial t}=-v_{z}\frac{{\partial c}_{i}}{\partial z}\pm r_{i}$$ 3

where *c*_*i*_ represents the concentration of the reactant or product *i*, *v*_*z*_ is the velocity in the *z* direction, and *r* is rate of reaction.

The general form of the rate law (reaction rate) for a bimolecular irreversible reaction is given by

$$r=kc_{1}c_{2}$$ 4

The reaction rate constant (*k*) follows an Arrhenius law, expressed as

$$k=k_{\mathrm{ref}}\exp\left(\frac{-E_{a}}{R}\times \left(\frac{1}{T_{f}}-\frac{1}{T_{\mathrm{ref}}}\right)\right)$$ 5

Here *k*_ref_ represents the reference reaction rate coefficient, *E*_a_ is the activation energy, *R* is the universal gas constant, and *T*_f_ and *T*_ref_ represent the feed temperature and reference temperature, respectively,

The energy equation accounts for the heat of reaction, diffusive flux, and heat exchange between the reaction side and the coolant. The energy balance equation is given by eq 6.

$$\rho C_{p}\frac{\partial T}{\partial t}=-v_{z}\rho C_{p}\frac{\partial T}{\partial z}\pm r\times \Delta H_{\mathrm{react}}+\mathrm{UA}(T_{c}-T)$$ 6

$$C_{p}=\underset{i}{\sum }x_{i}C_{pi}$$ 7

where *C*_*p*_ in the above reaction refers to the average specific heat capacity and *C*_*p,i*_ specific heat capacity of *i* ^th^ species, *x*_*i*_ is the mole fraction of each species, *T* and *T*_c_ represent temperature inside the tubular reactor and of the coolant, respectively. UA is the overall heat transfer coefficient, and Δ*H*_react_ is the reaction enthalpy. All relevant parameter values can be found in Table 1.

**Table 1:** Model Parameters, Physical Properties, and Initial Conditions

variables (unit)	values
length, *L* (m)	0.15
volume, *V* (m^3^)	1.32 × 10^–6^
UA (W/K)	100
Δ*H*_react_ (kJ/mol)	160
*C*_*p*,1_ (J/mol·K)	172
*C*_*p*,2_ (J/mol·K)	207.2
*C*_*p*,3_ (J/mol·K)	165.7
*C*_*p*,solvent_ (J/mol·K)	81.2
ρ (kg/m^3^)	800
benzylamine stock concentration, *C*_s1_ (mol L^–1^)	4
benzaldehyde stock concentration, *C*_s2_ (mol L^–1^)	4
gas constant, *R* (J K^–1^ mol^–1^)	8.314
residence time bounds, Res_*t*_ (min)	[0.5, 8]
equivalent ratio bounds, ER_1/2_	[0.1, 2]
temperature bounds, *T* (*K*)	[278, 330]

#### 2.1.2 Experimental Setup and Analytical Technologies

The experimental setup was designed to estimate kinetic parameters and validate the reaction mathematical model using inline spectroscopic measurements. The reaction was conducted in a microreactor system composed of coiled 1/16-in. stainless steel tubes. The reactor setup allowed for adjustable residence times between 0.5 and 8 min, ensuring near plug flow behavior throughout the experiments. Starting materials were delivered with high precision using an AZURA P 4.1s compact pump (Knauer, Germany). A detailed diagram of the setup is shown in Figure 2.

**Figure 2:**
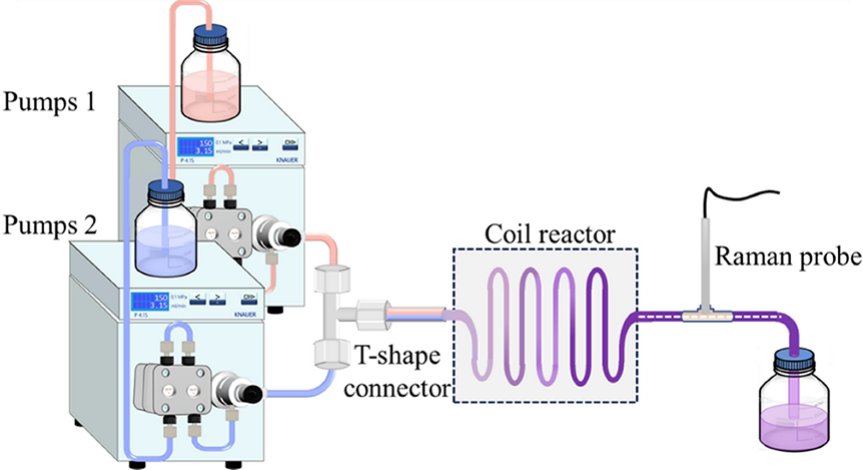
Reaction experimental set up for the proposed imine synthesis reaction in flow.

Real-time reaction monitoring was achieved using an inline Raman spectrometer (Raman Rxn2 analyzer, Kaiser Optical Systems, Inc.). Raman spectra were recorded in the 500–1900 cm^–1^ range with a spectral resolution of 4 cm^–1^. Spectra were acquired at 30-s intervals, with each spectrum accumulated over a 5-s duration. Key Raman peaks were identified at 1596 cm^–1^ (ring C=C stretching), 1639 cm^–1^ (C=N stretching), and 1684 cm^–1^ (C=O stretching). The recorded spectra revealed significant intensity changes during the reaction course. The C=O stretching peak at 1684 cm^–1^ decreased steadily and disappeared completely, indicating full conversion of the starting material. In contrast, the C=N stretching peak and the ring C=C stretching peak increased in intensity, signifying the formation of the imine product and the associated π-electron conjugation enhancement. These inline measurements provide valuable time-resolved data to support kinetic parameter estimation and validation the proposed reaction model.

### 2.2 Reinforcement Learning Agent

Reinforcement learning (RL) agents are designed to interact with their environment and autonomously choose the optimal behavior. The agent’s selection of a set of specific actions (i.e., reaction conditions) to be implemented in the environment is informed by the rewards received based on the previous actions and current circumstances captured by the states of the environment. Consequently, the agent receives a new reward or penalty which after combination with the prior knowledge informs the new sets of actions to be taken. Each sequential step involves interactions between the agent and the environment through state, action, and rewards (*r*_*t*_).^62^ Subsequently, throughout a sequence of recurrent episodes, the agent is instructed to maximize cumulative rewards (*G*_*t*_)­

$$G_{t}=\overset{\infty }{\underset{t=0}{\sum }}\gamma^{t}r_{t}$$ 8

where γ is the discount factor and signifies the importance of the future reward.

The primary objective of an RL algorithm is to identify and implement the best sequence of actions over an episode to maximize the accumulated reward. Thus, agents attempt to determine the optimal policy to maximize the expected return given by a state-value function (V-function) or state-action pair value function (Q-function).

Traditional methods for estimating value functions or policies in reinforcement learning (RL) typically rely on tabular approaches or linear function approximations. These methods work well for simple environments with discrete and small state-action spaces, as seen in techniques like Q-learning or temporal difference (TD) learning.^63^ However, when faced with complex or high-dimensional problems, such as those encountered in chemical reactions, these traditional approaches become impractical. The challenge arises from the difficulty of manual feature engineering, which involves selecting and designing relevant features from raw data. This process can be time-consuming, error-prone, and may lead to instability or divergence in the learning process, further complicating the model’s ability to generalize to new situations.

To address these limitations, deep learning methods were integrated with RL, leveraging deep neural networks (DNNs) to more effectively approximate value functions, policies, and models within the RL framework. DRL offers several key advantages over traditional RL.^64,65^ It excels in handling high-dimensional state and action spaces, thanks to the capacity of deep neural networks to represent intricate functions. DRL also automates feature learning from raw data, reducing the need for manual feature engineering and improving adaptability to complex environments. This capability enhances scalability and generalization, allowing DRL to perform well in diverse and previously unseen scenarios. Furthermore, DRL enables end-to-end learning, integrating perception and decision-making into a unified process, which improves overall performance and flexibility in tackling sophisticated problems.^59^

A deep deterministic policy gradient (DDPG), which is one of the family of DRL algorithm, is proposed to address the challenges associated with environments exhibiting continuous action spaces and high-dimensional and complex actions and achieve a nuanced exploration-exploitation trade-off.^58^ The proposed DDPG integrates value-based techniques like Q-learning with policy-based approaches such as policy gradient methods to deliver a robust framework for concurrently learning complicated behaviors. This integration allows DDPG to leverage the stability and robustness of value-based methods while benefiting from the flexibility and adaptability of policy-based approaches, enhancing learning efficiency in complex and continuous action environments.

#### 2.2.1 DDPG Framework and Network Design

The DDPG algorithm employs a sophisticated framework consisting of two key models: the actor and the critic. The proposed actor network architecture consists of three tiers: an input layer that receives the state as input, an output layer that generates the action, and hidden layers, which are two layers with 64 nodes each that record intricate interactions within the input. The critic network follows a similar structure but takes both the state and the selected action as input. After the input layer and several hidden layers, the concatenation layer and output layer are utilized to produce a single *Q*-value. This actor-critic network design offers a compromise between the RL agent’s ability to capture complex policy behaviors and the necessity for computational efficiency, thereby enabling stable training and practical deployment in reaction optimization tasks.^53,58^ While more complex network configurations could potentially enhance performance, they would come with increased computational costs, longer training times, and a higher risk of overfitting, which may not justify the performance gains in reaction optimization tasks where computational resources are a key limitation.^58^ Then, nonlinearity is introduced to both networks through nonlinear transformation or activation functions such as logistic, SoftMax, tanh or rectified linear unit (Relu). These functions allow neural networks to represent complex relationships between inputs and outputs, enabling them to approximate any continuous function and make them more efficient than linear transformations.^66^ The choice of ReLU activation function is primarily motivated by its computational efficiency, gradient stability, and established success in reinforcement learning tasks like DDPG.

After computations, error derivatives are computed backward and gradients backpropagated toward the input layer, allowing weights to be updated to optimize a loss function. A generalized architecture of the actor and critic network for DDPG agent is presented in Figure 3.

**Figure 3:**
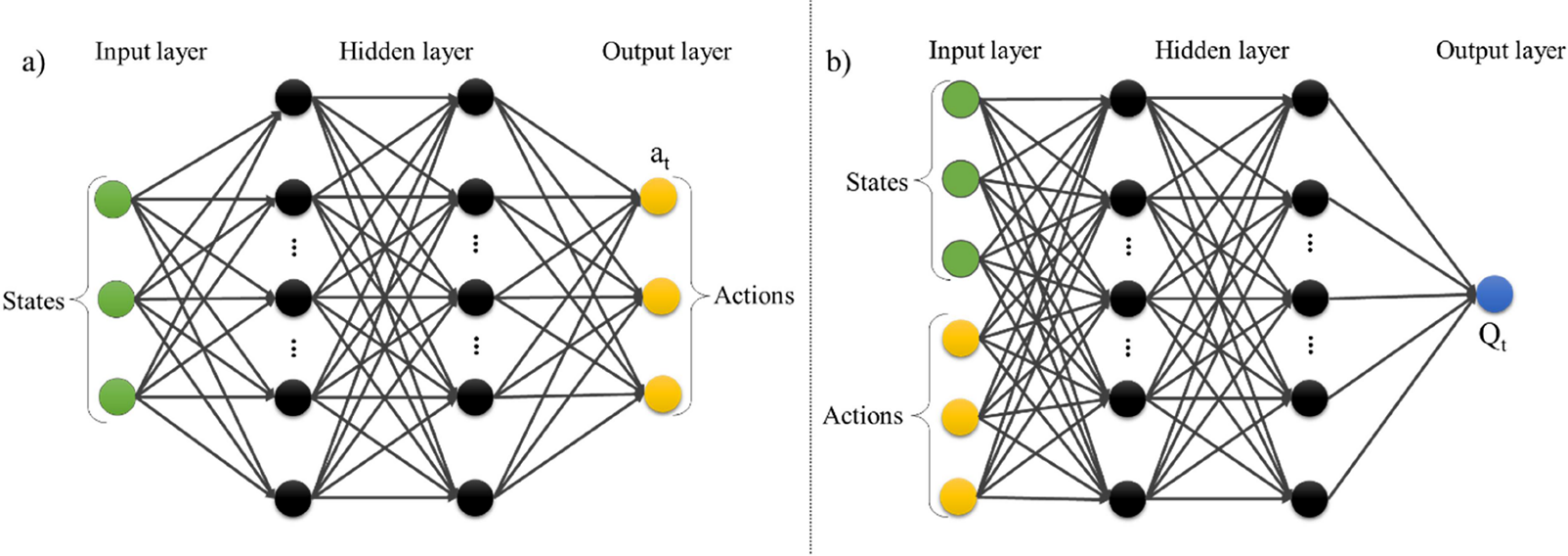
Deep neural network architectures of the proposed Actor (a) and Critic (b) models in the DDPG agent, each featuring two hidden layers with 64 nodes.

These actor and critic networks collectively guide the behavior of the RL agent in continuous action spaces. The actor is a policy network that receives the state as input and produces a precise continuous action, rather than a probability distribution of actions like DQN.^64,65^ Whereas the critic network evaluates the quality of actions chosen by the actor by estimating the state-action value function. Both networks have target networks, which are time-delayed copies of the actor and critic networks. They stabilize the learning process by acting as targets during updates.^63^

The actor network μ­(*s* | θ^μ^) is responsible for defining the current policy by deterministically mapping the current state of the environment to a specific action (reaction conditions variations). According to Lillicrap et al.,^58^ in off-policy algorithms such as DDPG, the exploration process can be treated separately from the learning process. Hence, the exploration policy with the addition of noise sampled from a noise process is given as follows:

$$a_{t}\left(s_{t}\right)=\mu \left(s_{t}|\theta_{t}^{\mu }\right)+N$$ 9

Here, *s*_*t*_ and *a*_*t*_ denotes the reactions conditions and their variations at time *t*, respectively. Noise ( N ) is introduced to the action to encourage exploration of the action space in the early stages of training. This is essential for balancing the exploration–exploitation trade-off, a fundamental aspect of RL. Exploration refers to the process of trying new actions or strategies to gather more information about the environment or solution space, enabling the agent to discover potentially better solutions that may not have been previously considered. In contrast, exploitation involves leveraging known information to make decisions that are expected to yield high rewards or optimal outcomes based on prior experiences or learning.^67^

After every iteration of the agent interaction with the environment, the network parameter (θ) is updated to optimize the probability that “good” behaviors will be sampled later. These transitions (*s*_*t*_, *a*_*t*_, *R*_*t*_, *s*_*t*+1_) are stored in the experience replay buffer. A minibatch of transitions (*N*) is sampled from the buffer for updating the actor and critic network.

To maximize cumulative rewards, the DDPG algorithm uses policy gradients to optimize policies by iteratively adjusting the parameters (θ^μ^). The gradient for the actor network is computed using the deterministic policy gradient theorem, which is given as

$$\nabla_{\theta^{\mu }}J\approx \frac{1}{N}\underset{i}{\sum }\nabla_{a}Q\left(s,a|\theta^{Q}\right){{|}_{s=s_{i},a=\mu \left(s_{i}\right)}\nabla_{\theta^{\mu }}\mu \left(s|\theta^{\mu }\right)|}_{s_{i}}$$ 10

In essence, the steps in eq 10 involve first computing the gradient of the action with respect to the actor’s parameters and then computing the gradient of the *Q*-value against the action. This dual-gradient strategy allows the actor network to be trained in a way that maximizes the expected *Q*-value, hence refining the actions it selects to achieve improved performance and enhancing the policy.^58,68^

The critic network *Q*(*s*, *a* | θ^*Q*^) evaluates the quality of the actions suggested by the Actor network and provides feedback by estimating the values (*Q*-value or reward) of the actions chosen by the actor.^64,65^ The evaluation of the *Q*-value is very critical for evaluating and refining the policy. To demonstrate the influence of various reaction conditions in this work, the *Q*-value function (*Q*_θ_) is presented as follows:

$$Q_{\theta }\left(s_{t},a_{t}\right)=E_{\theta }\left[\overset{\infty }{\underset{t=0}{\sum }}\gamma^{t}r\left(s_{t},a_{t}\right)\right]$$ 11

where *E*_θ_ denotes the expected value based on the policy, γ is the discount factor, and *r­(s*_*t*_ *, a*_*t*_) is the instantaneous reward associated with the actions *a*_*t*_ resulting in states *s*_*t*_. This formulation captures the long-term value of the state-action pair under the policy parametrized by θ. To practically compute and update these *Q*-values, the Bellman equation used, which provides the recursive relationship for the *Q*-value:

$$Q\left(s_{t},a_{t}\right)=r\left(s_{t},a_{t}\right)+\gamma \max Q\left(s_{t+1},a_{t}\right)$$ 12

The Bellman operator minimizes the TD error by reducing the difference between the Q function’s value before and after the update, estimating the expected value for the future state, *s*_*t*+1_. This error is usually calculated with the distinct target policy (actor) and value (critic) networks, each with unique parameters (θ^μ^′, θ^*Q*^′), to stabilize learning. Using the two-norm of this error, the critic loss is expressed as

$$L\left(Q^{\theta }\right)=\frac{1}{N}\underset{i}{\sum }{\left(y_{i}-Q\left(s_{i},a_{i}|\theta^{Q}\right)\right)}^{2}$$ 13

where *y*_*i*_ represents the target *Q*-value for the next state, computed using the target networks, which is defined as

$$y_{i}=r_{i}\left(s_{t},a_{t}\right)+\gamma Q^{\prime }\left(s_{i+1},\mu^{\prime }\left(s_{i+1}|\theta^{\mu^{\prime }}\right)|\theta^{Q\prime }\right)$$ 14

Minimizing the mean-squared loss between the target *Q*-value and the main *Q*-value (*Q*(*s*_*i*_, *a*_*i*_ | θ*^Q^*), as shown in eq 13, helps in optimizing the critic network approximation. This process ensures that the critic network accurately evaluates the quality of the actions, which, in turn, helps improve the actor network’s policy updates.

The target network of actor μ′(*s* | θ^μ^′) and critic *Q*′(*s*, *a* | θ^*Q*^′), are designed to gradually follow the learning of the main networks. This gradual update significantly improves the stability of the learning process by preventing large, abrupt changes that could hinder it. The target network weights are updated using “soft updates,” where a fraction of the main network weights is incorporated with a smoothing factor (τ)^58,69^ :

$$\theta^{Q^{\prime }}\leftarrow \tau \theta^{Q^{\prime }}+\left(1-\tau \right)\theta^{Q}$$ 15

$$\theta^{\mu^{\prime }}\leftarrow \tau \theta^{\mu^{\prime }}+\left(1-\tau \right)\theta^{\mu }$$ 16

The policy gradient of the actor network depends only on a proficient critic network. This suggests that any improvements made to the critic network will result in an instantaneous improvement in the quality of the actor network updates. Finally, the deployment of the DDPG algorithm is completed by training a policy network using a deterministic policy gradient (eq 10) and a Q-learning network by minimizing the error between the actor and target actor (eq 13). This combination of deep learning and an off-policy actor-critic network builds a powerful framework for effective training in high-dimensional state and action spaces, as shown in Figure 4.

**Figure 4:**
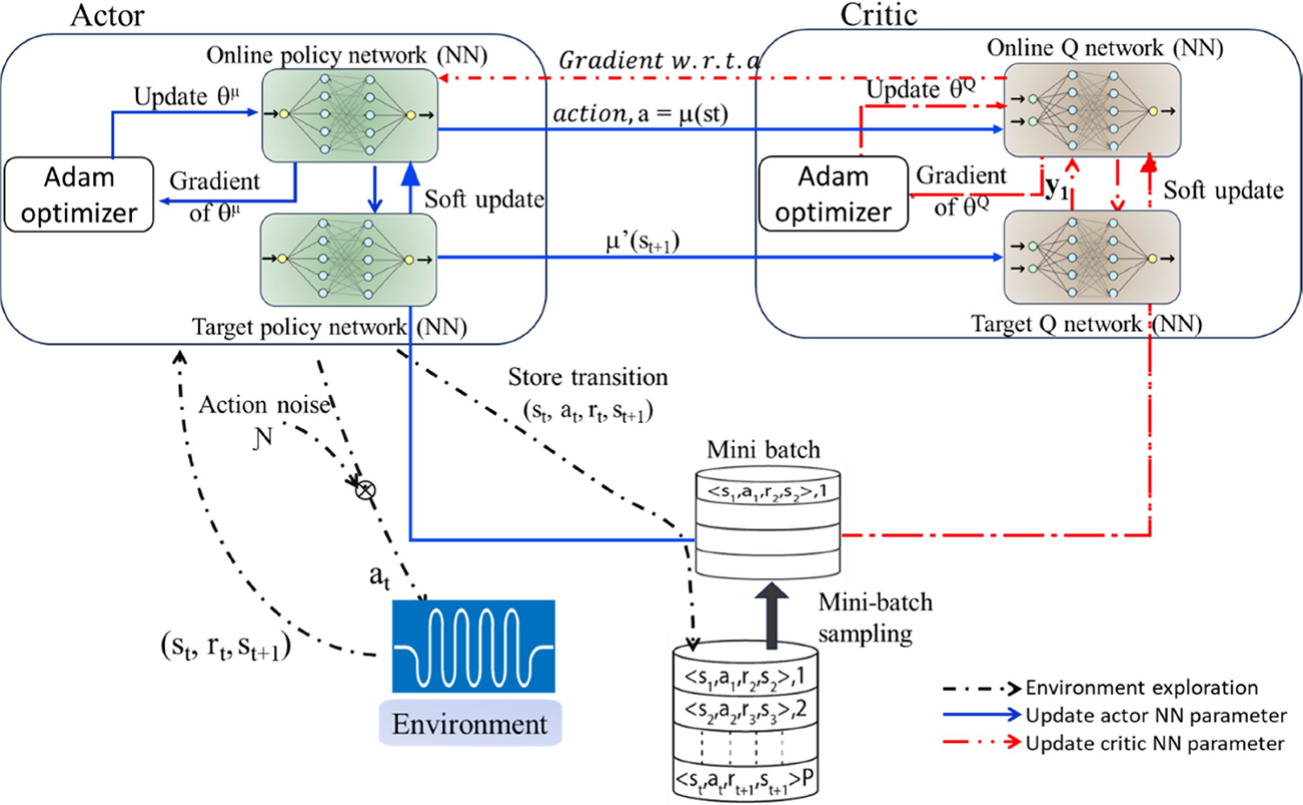
Framework of the DDPG RL agent.

The agent can be conceptualized as an optimization algorithm that aims to learn how to solve a set of objective functions. The state of the agent includes the current experimental conditions in the optimization process and any relevant features like gradients, previous conditions, and objective values. The action taken by the agent is the adjustment made to the current reaction conditions during the optimization process. The policy is the method used to determine which action to take based on the current state and the history of gradients, experimental conditions, and objective values. Learning the policy is learning the best way to update the experimental conditions. The RL algorithm ensures the agent maintains flexibility in solving the objective function without becoming overly specialized.

### 2.3 Reward Function

As discussed in the previous section, the rewards help the agent refine its actions and policies to converge on an optimal policy or solution. At every step of the episode, the agent receives a reward (*r_t_*) which by accumulating over all steps within an episode, helps to evaluate the total or cumulative reward that needs to be maximized. Here, the objective is to maximize the production of *n*-benzylidenebenzylamine, which is measured by the normalized concentration of the product. To achieve this, a hybrid reward function is proposed, as described in eq 17:

$$\begin{array}{c} c_{3,n}{\mathrm{norm}}_{c}=\frac{c_{3}-\min(c_{3})}{\mathrm{mix}(c_{3})-\min(c_{3})}\\ r_{t}=\{\begin{array}{cc} {\mathrm{norm}}_{c}+100 & \mathrm{if},c_{3}>1.6\\ {\mathrm{norm}}_{c} & \mathrm{else}\;\mathrm{if},1.2<c_{3}\leq 1.6\\ {\mathrm{norm}}_{c}-15 & \mathrm{else},c_{3}\leq 1.2 \end{array} \end{array}$$ 17

Here, *c*_3_ is the concentration of the product at the outlet of the reactor. Min (*c*_3_) and max (*c*_3_) are respectively minimum and maximum product concentrations experienced during training. Since the yield of *c*_3_ directly reflects the conversion rate with 100% selectivity (no side reactions or byproducts), the reward calculation aligns with yield computation. This means the reward function, based on the normalized concentration of c_3_, directly measures performance, simplifying the reinforcement learning process.

### 2.4 Hyperparameter Optimization

The training of RL agent involves both parameters and hyperparameters. Parameters, such as weights and coefficients, are internal values associated with DNN learned during training. In contrast, hyperparameters are external configuration settings of an agent that must be defined beforehand. Hyperparameter optimization is the process of systematically finding the optimal values for these external settings to maximize the performance. Hyperparameter settings play a critical role in shaping how the agent learns and adapts during training, influencing its performance and efficiency.^59,70,71^ In DRL, careful optimization is essential for optimizing learning speed, stability, and overall effectiveness. Techniques such as fANOVA have revealed that, depending on the environment, one or two hyperparameters may be significantly more critical than others,^72^ highlighting the need for more systematic and targeted optimization approaches. Traditional methods, such as OVAT tuning or grid search, often prove inefficient and costly, focusing solely on final hyperparameter settings without fully quantifying their impact throughout the training process. While OVAT methods are frequently used due to their simplicity and ease of implementation, they often fail to explore the hyperparameter space comprehensively and can be resource intensive. The goal here is not just to find good hyperparameter settings but also to quantify the effect of hyperparameter tuning. To overcome these limitations, Bayesian optimization has emerged as a sophisticated Hyperparameter Optimization (HPO) method. It models the objective function probabilistically, enabling a more targeted and efficient exploration of the hyperparameter space. Unlike the OVAT approach, which evaluates hyperparameters independently, Bayesian optimization considers the interactions between multiple hyperparameters simultaneously, leading to more informed and effective tuning.

One of the most widely used tools for using Bayesian optimization is Optuna, an open source hyperparameter optimization framework. Optuna leverages the Tree Parzen estimator to manage tree-structured search spaces and conditional parameters while utilizing kernel density function estimators for efficient optimization.^73^ The tool provides efficient search strategies, support for distributed computing, and automatic algorithm selection, making it a powerful tool for hyperparameter tuning. In this study, hyperparameters such as learning rates for actor and critic networks, noise exploration factors, discount factors, and smoothing factors were optimized. Performance metrics, including average reward, convergence time, and policy stability, were used to evaluate the effectiveness of each method. This work compares the performance of traditional OVAT tuning with Bayesian optimization, implemented using the Optuna framework, to identify which approach yields superior results in DRL tasks.

### 2.5 Training and Refinement

The training performance of any RL agent is strongly dependent on the exploration and exploitation trade-off. A RL agent is trained to find the optimal solution as fast as possible to minimize the experimental cost. However, selecting a solution too early without full exploration is not a good approach as it may leads to either unsuccessful training or suboptimal or local solutions. To achieve effective training, an agent must explore thoroughly the operating/decision space to identify optimal policies, in a cost-effective manner, and avoid suboptimal solutions. Several algorithms can be used to enhance exploration by adding randomness or noise to the actions, states, or directly to the weights of the actor network. When selecting actions, noisy policies add randomness directly to the decision-making process while ε-greedy policies probabilistically balance exploration for new actions and exploitation of known ones.

Noise based exploration was suggested by Lillicrap in their introductory paper for continuous action.^58^ As given in eq 9, the noise ( N ) can be tailored to suit the characteristics of the environment by adjusting its variance, exploring different noise distributions, and making it adaptive to the agent’s performance and environment conditions. The proposed method employs the Ornstein–Uhlenbeck (OU) process, a stochastic process introduced by Uhlenbeck and Ornstein^74^ and later used by Lillicrap et al.^58^ to generate time-dependent temporally correlated noise. OU noise is calculated independently for each action, as follows:

$$\chi_{a_{t}}=\chi_{a_{t-1}}+\theta (\mu -\chi_{a_{t-1}})\cdot dt+\sigma \sqrt{dt}\cdot N(0,I)$$ 18

Here, χ_*a*_*t*__ is the current value of the noise, θ suggests the rate at which noise returns to the mean, μ represents the long-term mean, and σ is the volatility of the noise. The mean serves as a central tendency or baseline value for the noise over time N(0,I) represents a random variable sampled from a standard normal distribution The OU process introduces autocorrelation in the noise, which helps the agent explore the action space more effectively.

In ε-greedy, the exploration-exploitation trade-off is achieved by randomly selecting actions at the beginning of the training and more purposefully toward the end of training. The agent computes epsilon (ε), as follows:

$$\varepsilon =\varepsilon_{\min}+\frac{\left(1-\varepsilon_{\min}\right)}{e^{-\lambda -N_{t}}}$$ 19

where ε_min_ is the lowest probability of choosing a random action at the end of the training. The parameter λ, which governs the rate of exponential decay, gives the rate of exploration decline over time. As the number of training iterations (*N*_*t*_) increase, the exponential decay of ε indicates a decreasing likelihood of selecting random actions. This decay mechanism ensures that the agent proceeds from exploratory activity to the gradual exploitation of learned information, hence improving its decision-making capabilities. By using exponential decay to adjust ε intelligently, the agent begins with high probability of exploring new actions with limited knowledge and discover various possible strategies. As training progresses, ε decreases, leading the agent to exploit best known actions more frequently. This balance allows the agent to collect comprehensive information and enhance decision-making based on accumulated knowledge. Additionally, this improves the agent’s learning trajectory, integrating new information and prior learning for more informed decisions in complex situations.

## 3 Results and Discussion

In this section, the methodology and RL algorithms described in the previous section are implemented. The simulations were conducted in Python version 3.9. The mathematical model described earlier (eqs 3–6), representing the imine synthesis in flow, is used as the environment to train the proposed DRL agents and validate and test the proposed architectures and methods. The model is simulated using the initial conditions and variable values outlined in Table 1. The method of lines is employed to convert the partial differential equations into ordinary differential equations (ODEs), which can be solved numerically. In the current case, the resulting ODE system is solved in Python using the ‘solve_ivp’ function, which addresses initial value problems with stiffness. The solver employs a backward differentiation formulas method, a robust technique designed to efficiently solve stiff equations. The agent interacts with the reactor model (environment) continuously by changing the manipulated variables (actions) and collecting the states to evaluate the rewards. A complete list of Python packages used is provided in Section A of the Supporting Information (SI).

Prior to the self-optimization efforts, a parameter estimation procedure was conducted based on collected experimental data. This is followed by a comprehensive validation and testing of the DDPG and reaction network setting. The impact of hyperparameters on the training procedure and outcomes is demonstrated through systematic parametric analysis. At the end, the performance of the DRL approach is compared against the state-of-the-art techniques such as SnobFit and Nelder–Mead.

A DRL agent was designed based on the proposed actor and critic network architecture as discussed in the previous section. The DDPG agent was trained based on a maximum number of episodes of 100, each episode consisting of 10 decision/action steps. The replay buffer size is kept at 5000 to store training experience, which allows the algorithms to learn from a more diverse set of experiences.

### 3.1 Estimation of the Model Parameters

To build a reliable model able to capture or represent the RL environment, several model parameters must be identified based on a set of experimental data. Here the kinetic parameters, namely the activation energy (*E*_a_) and the rate coefficient *k*_ref_, which are critical to predict how the reaction behaves under various conditions and consequently allowing systematic optimization of the reaction process, are identified from the experimental data. Accurate determination of these parameters ensures that the mathematical model, used as a training environment for the DRL agent, aligns closely with real-world outcomes. The remaining model parameters (e.g., UA, Δ*H*_react_) were fixed at nominal values obtained from the literature (Table 1).

The results shown in Figure 5 represent the concentration profiles over time obtained under four different temperatures (288, 298, 313, and 318 K). The experimental data capturing the concentration profiles of the reactant (benzaldehyde) and product (imine) were used for the parameter estimation procedure based on the minimization of the sum of the squared errors. The optimal kinetic parameters are summarized in Table 2. As shown in Figure 5, the model demonstrates excellent prediction capabilities with respect to the four different experiments. Additional details are provided in Section B of the Supporting Information (SI).

**Figure 5:**
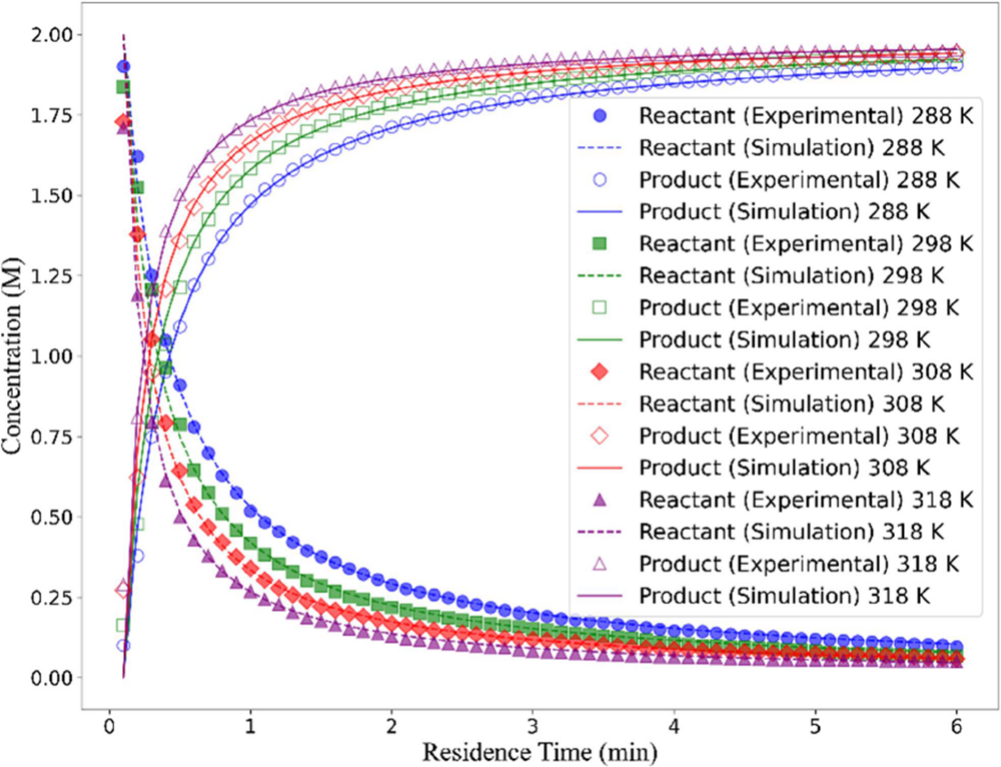
Model predictions vs experimental data (a) Concentrations of the product and reactant (benzaldehyde) at the reactor outlet, at different temperatures.

**Table 2:** Optimal Estimates of the Kinetic Parameters of the Imine Synthesis

parameter	optimal value
activation energy, *E*_a_ (kJ mol^–1^)	21.57
reaction rate coefficient *k*_ref_ (L mol^–1^ min^–1^)	0.413

### 3.2 Tuning of the DRL Hyperparameters

In DRL, the performance of an agent is strongly dependent on the choice of the hyperparameters. In this section, we present and compare the results associated with the hyperparameter optimization using an advanced technique, namely Bayesian optimization via Optuna, against a tuning strategy based on the traditional trial-and-error methods. Both approaches were evaluated under identical conditions, including the same environments, network architectures, and performance metrics. Here, the emphasis is on five key hyperparameters, namely the learning rates for the actor and critic networks, discount factor, target network update rate, and noise addition, which significantly impact the agent’s decision-making, stability, and gradient descent efficiency.

The learning rates of the actor and critic networks are indeed critical hyperparameters in DRL algorithms. The convergence performance of the proposed algorithms with various learning rates is illustrated in Figure 6. The figure clearly shows that both very low and very high learning rates result in poor convergence. The learning rate determines the magnitude of the parameter adjustments during training, directly influencing how quickly or stably the model learns. When the learning rate is too high, parameter adjustments become overly large, often destabilizing the training process. This can lead to oscillations or divergence by overshooting the optimal solution, even when using adaptive optimizers like Adam, which dynamically adjust step sizes based on gradient information. High learning rates are particularly prone to causing instabilities, as the excessively large parameter changes hinder convergence. Conversely, very low learning rates result in infinitesimal parameter changes, significantly increasing the number of iterations or episodes required for training and resulting in slower convergence. This slower progress can also increase the likelihood of the model becoming trapped in suboptimal solutions. Therefore, carefully selecting an appropriate learning rate is crucial, as it governs the scale and stability of parameter refinement throughout the training process. Based on the observed performance, the optimal learning rates for updating the actor and critic networks are α_actor_ = 0.001 and α_critic_ = 0.002, respectively. These values help achieve a good trade-off between stability and convergence speed.

**Figure 6:**
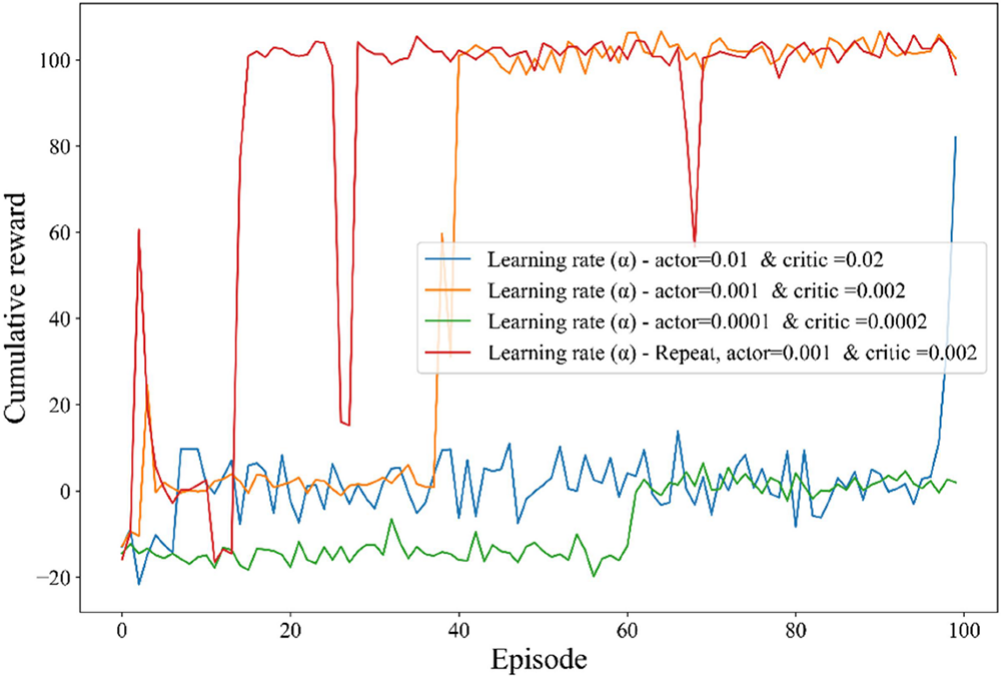
Convergence of training noisy action policies at different actor and critic learning rates.

The exploration noise parameter (σ_e_), which introduce randomness into the actions taken by the agent, are crucial for effective exploration within the decision space. In this study, the impact of the exploration noise parameter was evaluated within the range of 0.0001 to 0.5. This range was chosen to be very wide to cover a broad spectrum of exploration behaviors, from minimal to extensive noise. The results of this investigation are presented in Figure 7. When σ_e_ is set too low (e.g., between 0.0001 and 0.005), the agent experiences minimal randomness, leading to inadequate exploration, premature convergence to suboptimal policies, and a tendency to become trapped in local optima. Conversely, larger values of σ_e_ result in more important noise distributions, encouraging the agent to explore a broader range of actions. However, the impact on performance varies depending on the specific value of σ_e_. For example, with a higher value like σ_e_ = 0.5, the exploration noise is significant, leading to substantial randomness in the agent’s actions. This excessive exploration can cause unstable performance, with cumulative rewards fluctuating between 20 and 40, as the agent struggles to maintain a stable policy due to the erratic behavior induced by the high noise level. Therefore, as shown in Figure 7, the observed optimal value of σ_e_ lies around 0.01, where exploration is sufficient to discover a broad set of potential policies without disrupting the stability and efficiency of the learning process.

**Figure 7:**
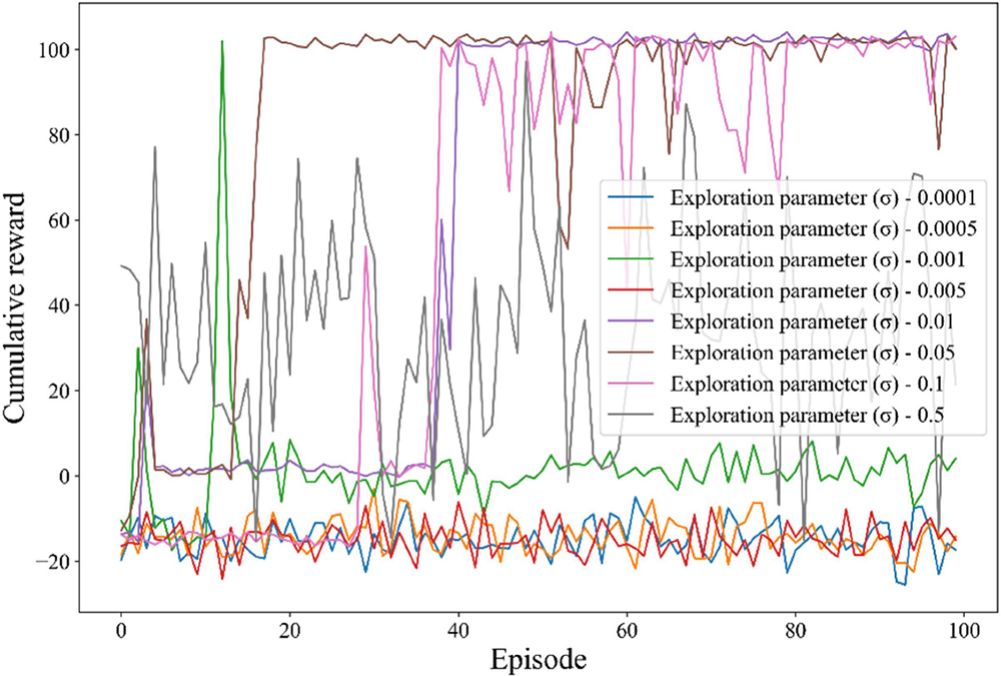
Training performance and convergence at different values of the noise exploration parameter (σ_e_).

The discount factor, denoted as γ, is a critical hyperparameter in the DDPG framework, as it determines the weight given to future rewards relative to immediate rewards, thereby influencing the balance between long-term and short-term learning and decision-making. In our study, we examined discount factors ranging from 0.001 to 0.999 to assess their impact on the agent’s learning process and overall performance. When γ is less than 0.7, the agent tends to prioritize short-term rewards, which can lead to more stable but potentially suboptimal learning curves. This gratification of immediate rewards impairs the agent’s ability to consider long-term benefits, resulting in poor training responses and limited overall performance, as illustrated in Figure 8. Conversely, a high discount factor, here above 0.9, enhances the agent’s resilience by mitigating its sensitivity to abrupt changes in the environment. This leads to improved stability in learning and better convergence, as the impact of noisy rewards is reduced. These findings suggest that a discount factor (γ) within the range of 0.9–0.999 provides a reasonable balance. By adopting values within this range, it, further stabilizes the learning process and places greater emphasis on long-term benefits, thereby improving overall learning performance.

**Figure 8:**
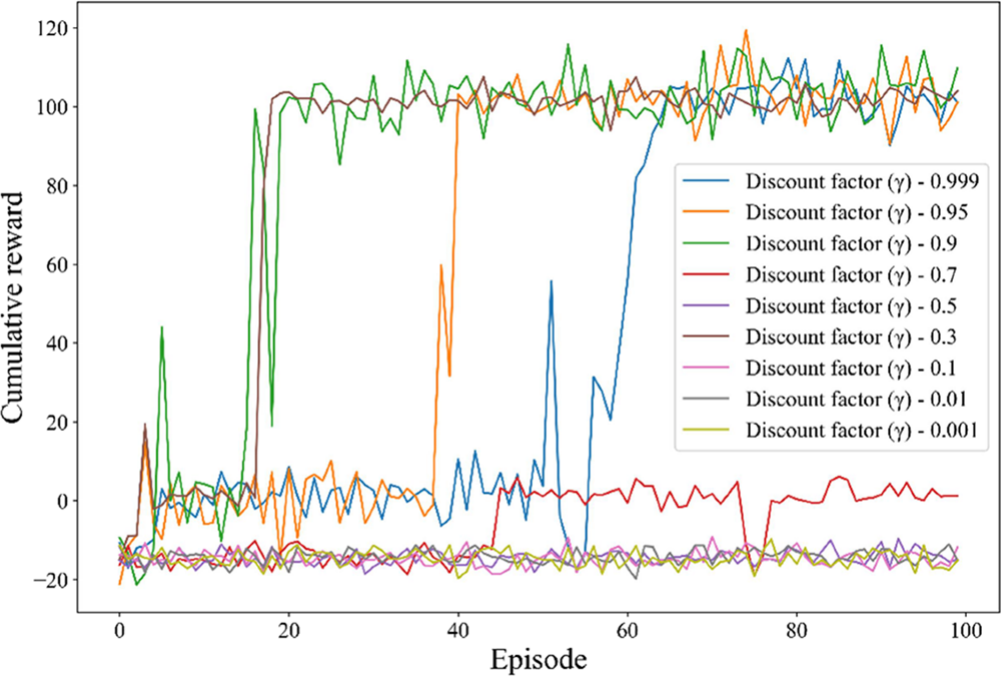
Training convergence under different discount factors.

The smoothing factor (τ) is also a critical parameter that controls the rate at which the parameters of the target network are updated relative to those of the main (or online) network. As discussed in Section 2.2.1, the main actor and critic networks are updated directly during training, whereas the target network undergoes a slower update using a soft update rule. This gradual update helps stabilize the learning process by preventing abrupt changes in the target values. As illustrated in Figure 9, varying τ values from 0.0001 to 1 significantly impacts the cumulative rewards achieved by the agent over the specified episodes. Specifically, higher τ values, such as 1 and 0.1, cause the target network’s parameters to more closely track those of the main network, resulting in faster updates. Although this can accelerate the learning process, it may also introduce instability if τ is set too large, as the target network may react too quickly to changes in the main network. Conversely, lower τ values, such as 0.001 and 0.0001, result in slower updates to the target network’s parameters, enhancing stability by reducing rapid fluctuations and oscillations. However, this comes at the cost of slower adaptation to changes in the main network, which could hinder learning. The observed performance trends in the graph underscore the importance of selecting an optimal smoothing factor that balances responsiveness and stability. An updated range of 0.001 to 0.01 emerges as a promising trade-off, allowing the agent to effectively adapt to changes in the environment while ensuring robust performance. Notably, the τ value of 0.01 demonstrates a good compromise, yielding substantial cumulative rewards while maintaining a stable learning trajectory. The observed performance trends illustrated in Figure 9 underscore the importance of selecting an optimal smoothing factor that balances responsiveness and stability. These findings highlight the critical role of the smoothing factor in shaping the learning dynamics of RL agents, emphasizing the need for careful hyperparameter tuning to enhance overall performance.

**Figure 9:**
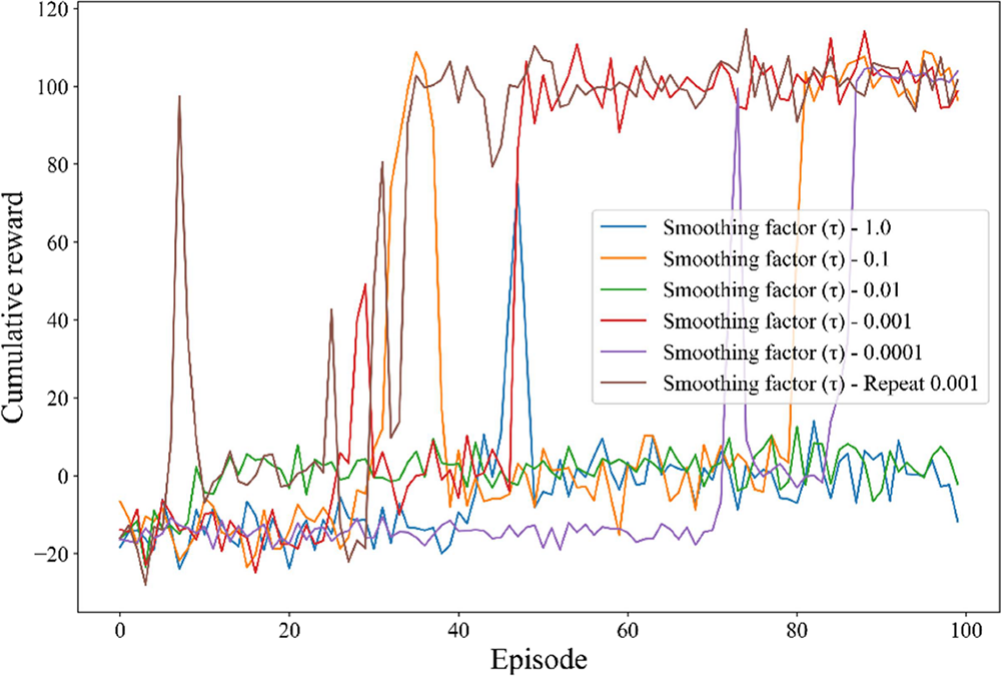
Smoothing factor (τ) influence on cumulative rewards under noisy environments.

All the optimized hyperparameters, including the discount factor γ, the smoothing factor τ, the learning rates for the actor and critic networks, and the exploration noise parameter σ_e_, are summarized in Table 3. The results reveal notable differences between the two approaches, highlighting the impact of systematic optimization on hyperparameter selection. A key advantage of Bayesian optimization is its ability to tune several or all hyperparameters simultaneously, whereas the trial-and-error method typically adjusts one parameter at a time, making it a more time-consuming and less efficient process. One notable observation is the significantly lower critic learning rate (α_critic_) achieved through Bayesian optimization (0.00043) compared to trial-and-error tuning (0.002). A lower learning rate for the critic network often enhances stability in DRL by preventing large updates that could destabilize training. Similarly, the smoothing factor (τ) is optimized to a much smaller value (0.0004) through Bayesian optimization, which suggests a more conservative update strategy for target networks, potentially leading to more stable learning. Another important difference is seen in the exploration noise parameter (σ_e_). Bayesian optimization results in a significantly higher value (0.1019 vs 0.01), indicating a preference for increased exploration during training. This suggests that Bayesian optimization identifies a balance between exploration and exploitation that may not be as easily achieved through manual tuning. The discount factor (γ) and actor learning rate (α_actor_) show smaller differences, with Bayesian optimization yielding slightly higher values (0.961 vs 0.95 for γ and 0.002 vs 0.001 for α_actor_). These adjustments may contribute to better long-term reward optimization and more effective policy updates.

**Table 3:** Ranges of DDPG Agent Training Hyperparameters and Their Optimal Values Obtained through Trial-and-Error and Bayesian Optimization

		optimal value	
hyperparameter	hyperparameter predefined range	trial-and-error	Bayesian optimization
critic learning rate (α_critic_)	0.0002–0.02	0.002	0.00043
actor learning rate (α_actor_)	0.0001–0.01	0.001	0.002
discount factor (γ)	0.001–0.999	0.95	0.961
exploration noise parameter (σ_e_)	0.0001–0.5	0.01	0.1019
smoothing factor (τ)	0.0001–1.0	0.01	0.0004

The computational cost of the different tuning strategies is also key. As anticipated, the Bayesian optimization demonstrates clear advantages over trial-and-error. As shown in Table 4, Bayesian optimization completed 100 trials across the considered 5 hyperparameters in approximately 11.47 h. In contrast, trial-and-error required 7 h to complete only 30 trials to deliver suboptimal performance. Another way to establish a reliable comparison between the Bayesian optimization and trial-and-error consists of running the latter under similar numbers of trials (i.e., 100 trials). As expected, the extended trial-and-error approach comes with an even higher computational cost without guaranteeing optimal performance. It is worth mentioning that the computational time does not increase linearly with the increased number of trials in all cases because changes in any given hyperparameter have a different impact on the training features and performance and consequently on the inherent computational cost. In essence, the training performance and the computational cost have different sensitivities to changes or increments in the hyperparameter values.

**Table 4:** Computational Time Associated with the Hyperparameter Tuning Based on Trial-and-Error vs Bayesian Optimization

HPO method	No. of trials	No. of hyperparameter	computational time
Bayesian optimization	100	5	∼11.47 h (41,292 s).
trial-and-error	30	5	7 h (25,200 s).
trial-and-error (extended)	100	5	∼2 days (∼172,800 s)

Overall, the results demonstrate that Bayesian optimization can explore a broader search space and fine-tune hyperparameters more effectively than the trial-and-error method. These optimized values could potentially lead to improved convergence speed, training stability, and final policy performance. Table 5 provides additional parameters relevant to the overall design and training process of the DDPG algorithm.

**Table 5:** Additional Parameters and Settings of the DDPG Agent

parameters	values
episode	100
iterations per time step	10
optimizer exploration policy	OU-noise
optimizer	Adam
batch size	64

### 3.3 Training Performance Using ε-Greedy and Action-Noise Policy

In this part, we investigate the training performance and inherent exploration strategy of the DDPG agent discussed in section 2.5. The action-noise policy was evaluated using the set of hyperparameters optimized using Bayesian optimization as discussed in the previous section, with the training performance illustrated in Figure 10. Figure 10a depicts the agent’s exploration performance within the decision space, transitioning to exploitation as training episodes progress. The DDPG agent’s training starts as shown by the purple dots, gradually converging to the optimal reaction conditions (red dots). Figure 10b illustrates the agent’s learning dynamics associated with the optimization of product concentration. The consistent initialization yet varied trajectories indicate the stochastic nature and complexity of the optimization process. The color gradient, representing product concentration, demonstrates the agent’s adaptive decision-making as it refines its policy to achieve optimal reaction conditions over successive training episodes. Figure 10c demonstrates that the DDPG agent, based on the hyperparameters optimized via Bayesian optimization, exhibits the best training and convergence performance toward the optimal operating conditions of the imine synthesis reaction. Initially, the agent explores the decision space during the first 40 training episodes, subsequently shifting to exploitation guided by the best policy. Notably, the training persists beyond the 50^th^ episodes, as indicated by the cyan-colored dots in Figure 10a, demonstrating the agent’s continued learning and adaptation to optimize performance. This extended training period allows the DDPG agent to further refine its policy and gain deeper insights into the system’s dynamic behavior based on finely tuned actions. The training is ultimately achieved after reaching the maximum number of episodes, marked by the red dots at the 100^th^ episode, or when the moving average of cumulative rewards stabilizes, indicating convergence to the optimal solution. This comprehensive analysis underscores the DDPG agent’s capability to navigate the decision space effectively, balancing exploration and exploitation to optimize performance of the imine synthesis reaction.

**Figure 10:**
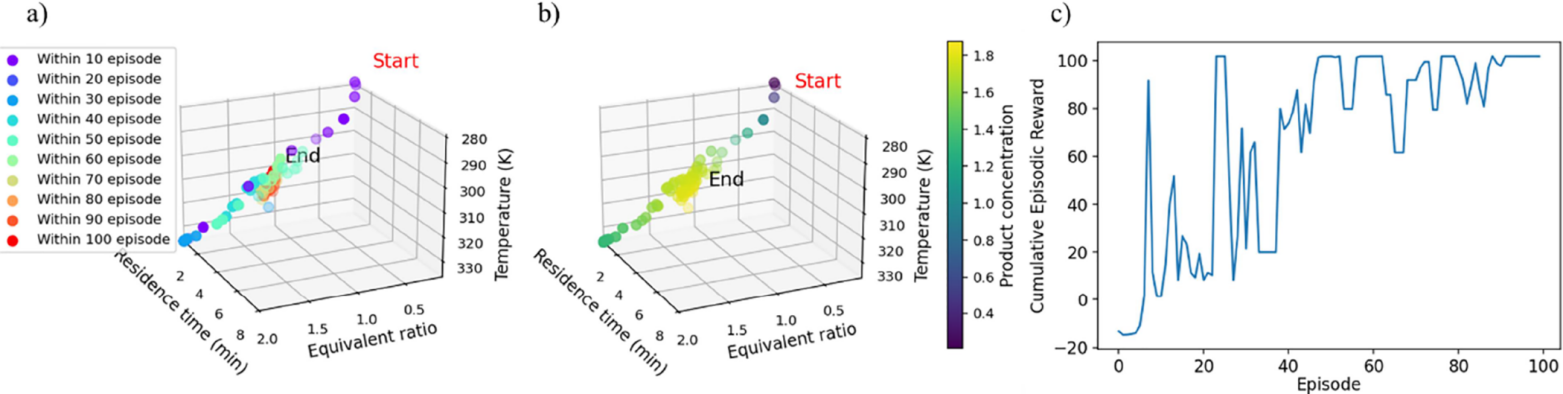
Training performance of noisy action policy at optimized hyperparameter.

Figure 11 shows the detailed training performance of the DDPG agent over the iterative interactions with the environment. The agent’s learning process begins with the initial actions selected randomly for each episode to explore the decision space. These initial action values, such as residence time, molar equivalent ratio, and reactor temperature, serve as a baseline from which the agent iteratively adjusts its decisions based on feedback (rewards) received from the environment. During early training episodes, the agent’s actions received low rewards, indicating the exploratory phase, which is critical for the agent learning process. Over time, the agent progressively converged toward higher cumulative rewards and associated optimal conditions. By episode number 28, the agent began to refine its actions, leading to increased cumulative rewards and inherently higher product concentrations. This behavior also captures a shift from exploration to exploitation of the learned strategies. Specifically in episode 40, molar ratio and temperature sequentially decreased from 1.7 to 1.4 and from 338 to 319 K, respectively, to increase the product concentration. Additionally, the residence time approached its optimal value during this phase, further contributing to the agent’s improved performance. As the training progressed, the agent continued exploring and gradually converged toward optimal conditions, as evidenced by the stabilization of the values of the decision variables in later episodes. For instance, actions between episodes 63 and 100 are finely tuned to continuously produce maximum productivity (more than 1.8 mol/lit), which suggests that during these first 63^rd^ episode, the agent was mainly performing exploitation to deliver global optimal reaction conditions with the help of the additionally collected knowledge from the environment (i.e., the imine synthesis reactor).

**Figure 11:**
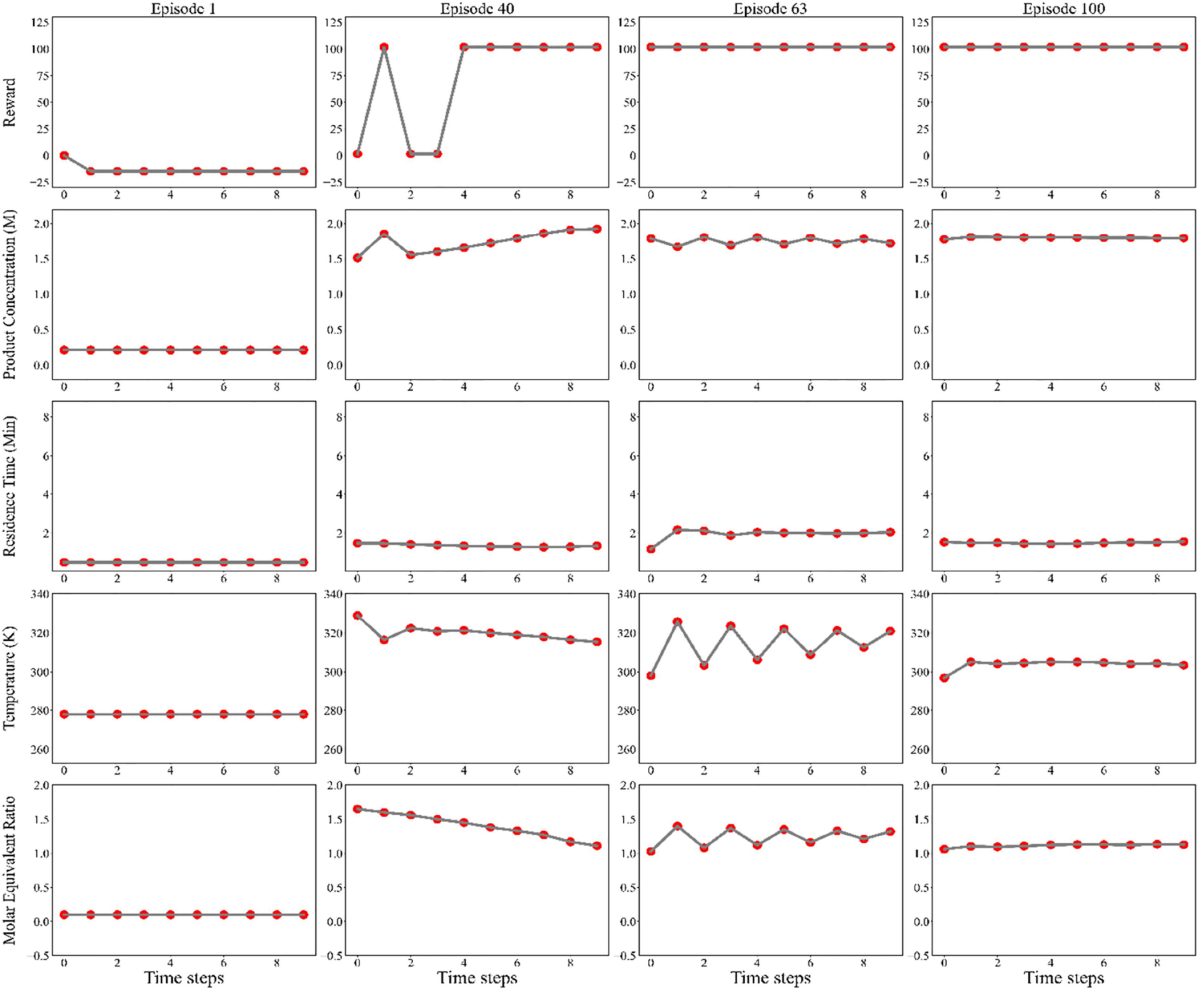
Evolution of the sequences of the decision variables (actions) implemented by the agent over the 1st, 40th, 63rd, and 100th training episodes. (Red dots indicate the moment at which the agent takes actions.)

The performance of the DDPG agent is evaluated under a noisy action policy and compared with the ε-greedy policy to gain insight into the agent’s resilience under various conditions, as explained in detail in section 2.5. Additionally, this allows a comprehensive assessment of the convergence rates, effectiveness, and environmental adaptation, offering insightful information about the fundamental processes and guiding the best line of training action in complex systems. The training performance of the ε-greedy policy is shown in Figure 12a,d,g, whereas Figure 12b,e,h depicts the corresponding decision spaces that the agent explored, and Figure 12c,f,i emphasizes the product concentration. Initially, as shown in Figure 12a–c, the DDPG agent trained under the ε-greedy policy with the same set of hyperparameters and setting (Tables 3 and 5) required additional episodes and significant simulation time to achieve convergence. From Figure 12a, it can be observed that the agent engaged in exploration for the first 60 episodes, as indicated by unstable and low episodic rewards, reflecting its attempt to learn the environment’s dynamics. However, as shown in Figure 12b,c, the agent explored only a limited section of the decision space before transitioning to the exploitation stage. This shallow exploration, coupled with the absence of convergence to specific optimal actions under the current conditions and settings, suggests the need for further improvements. Addressing this limitation may require additional data, either through increasing the number of episodes or the number of actions/steps per episode, to encourage deeper exploration of the environment representing the imine synthesis reaction.

**Figure 12:**
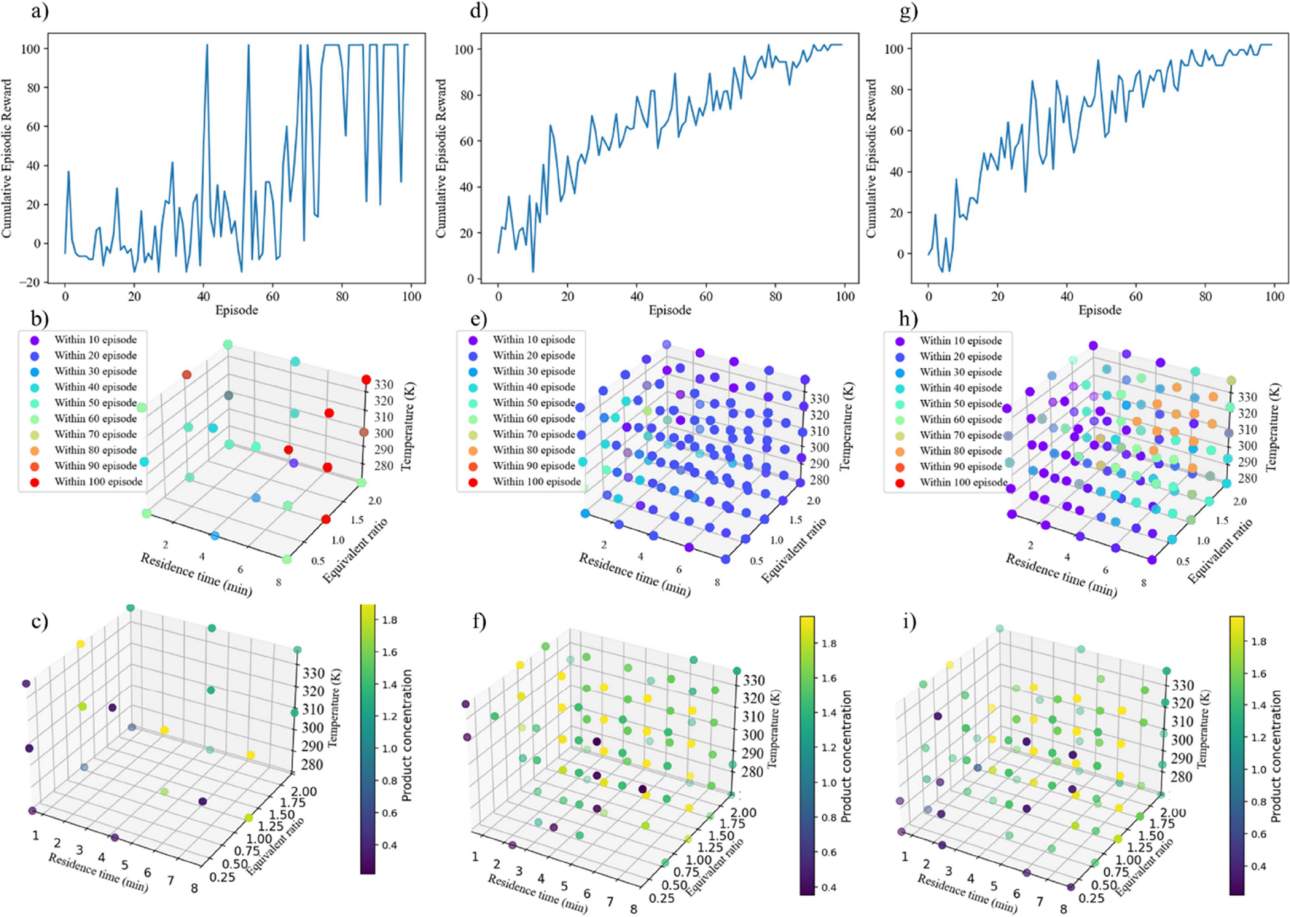
Training performance of the DDPG agent based on the optimized hyperparameters for the ε-greedy policy with batch sizes of 64 and 10 steps (a–c), 64 and 40 steps (d–f), and 128 and 40 steps (g–i), respectively.

To mitigate the observed shallow exploration, the number of steps per episode was increased from 10 to 40. This adjustment enabled the agent to evaluate a greater number of actions per episode, thereby generating more data points and enhancing its exploration capability. As shown in Figure 12d–f, the increased number of steps facilitated improved exploration, resulting in smoother and more stable training performance with higher cumulative rewards. Importantly, this approach reduced the need for additional episodes, though it came at the cost of increased computational demand. To further enhance training stability, the batch size was increased from 64 to 128. This modification allowed the agent to sample a larger number of experiences from the replay buffer at each training iteration, leading to more stable updates. As shown in Figure 12g–i, this adjustment resulted in smoother training curves and a more comprehensive exploration of the decision space. Ultimately, these changes enabled the agent to achieve improved and consistent performance, despite the trade-off of increased computational costs. The smoother training and more thorough exploration led to more effective exploitation of the learned policies, ensuring the agent could more reliably reach optimal conditions within the environment over time.

### 3.4 Adaptive Hyperparameters Tuning

The previous sections highlighted the performance of DDPG agents based on different hyperparameter tuning/optimization strategies. As clearly shown, the agent performance was significantly enhanced using the Bayesian hyperparameter optimization approach. However, fixing the hyperparameter values for the entire training duration may impose limitations due to the conflicting and competing nature of certain parameters, which require inherent tuning compromises. These trade-offs can affect the overall performance of the RL agent, as many of the parameters will be given suboptimal values, resulting in a poor training performance. To overcome these limitations, an adaptive or dynamic hyperparameter tuning will be investigated to deliver a more effective solution. Adapting the parameter values during training may enable the agent to maintain flexibility, fostering efficient exploration in the early stages while gradually emphasizing exploitation as learning progresses.

An effective adaptive tuning approach is employed to update the hyperparameters during training as described in eq 20 below. The method allows the implementation of a dynamically adjusted real hyperparameter value as opposed to methods based on discrete values, enhancing the adaptability of the proposed methodology.^75^

The hyperparameter values are dynamically adjusted over the training stages based on the equation below.

$${\mathrm{HP}}_{t}=\max({\mathrm{HP}}_{\max}-\alpha \times A_{t},{\mathrm{HP}}_{\min})$$ 20

In the equation above, HP_*t*_ represents the dynamically adjusted hyperparameter at time *t*, bounded by HP_max_ and HP_min_. α is the decay rate which ensures a smooth transition from higher to lower values as training progresses. *A*_*t*_ a progress or adaptation metric (*A*_*t*_) which depends on the total number of steps per episode (*N*_total_), the current episode (Ep_*t*_) and the current training step (*N*_*t*_). *A*_*t*_ is defined as

$$A_{t}={N_{\mathrm{total}}}^{*}{\mathrm{Ep}}_{t}+N_{t}$$ 21

During early training stages, higher hyperparameter values, closer to the maximum allowed value (HP_max_), are implemented as the adjustment term relating to the number of episodes and steps (*A*_*t*_) (eq 21) is still very small. As a result, the agent undergoes broader exploration. As the training progresses, the term *A*_*t*_ (in eq 21) increases and consequently the hyperparameter values gradually decrease and converge toward their minimum values (HP_min_). This gradual reduction ensures a smooth transition from exploration to exploitation and enhances convergence toward the global optimal solutions.

This dynamic framework allows the agent to adapt its learning process, ensuring an optimal balance between exploration and exploitation throughout training. To demonstrate the capabilities of the adaptive hyperparameters tuning, two critical parameters will be dynamically tuned, namely the exploration noise and learning rates. The use of adaptive exploration noise, as opposed to fixed noise, delivers improved training performance, as shown in Figure 13a compared to Figure 10a, evidencing enhanced exploration and the agent’s capability to identify the global optimal conditions. At the beginning of training, higher levels of noise introduce substantial randomness, encouraging broad exploration of the decision space. During this phase, the agent actively explores the reaction decision space, taking sequential steps toward identifying the optimal conditions, shown in Figure 13a,b. As the agent becomes more confident in its learned policy, the noise is systematically reduced, facilitating a smoother transition from exploration to exploitation. This shift is reflected in the cumulative rewards, which initially show strong exploration up until approximately the 40^th^ episode. Following this, the agent begins to converge toward optimal reaction conditions, as evidenced by the clustering of points in the decision space toward the “End” point in Figure 13a,b, and, ensuring maximum cumulative rewards as it focuses on exploiting its learned strategy. Based on the convergence observed in the decision space (Figure 13a,b) and the stabilization of cumulative episodic reward (Figure 13c), the agent demonstrates effective learning throughout the training period. However, the distribution of data points within the decision space suggests that the extent of exploration may be insufficient to definitively conclude convergence toward a global optimum. To encourage further exploration and potentially identify superior reaction conditions, it is beneficial to gradually lower the learning rate for both the actor and critic networks.

**Figure 13:**
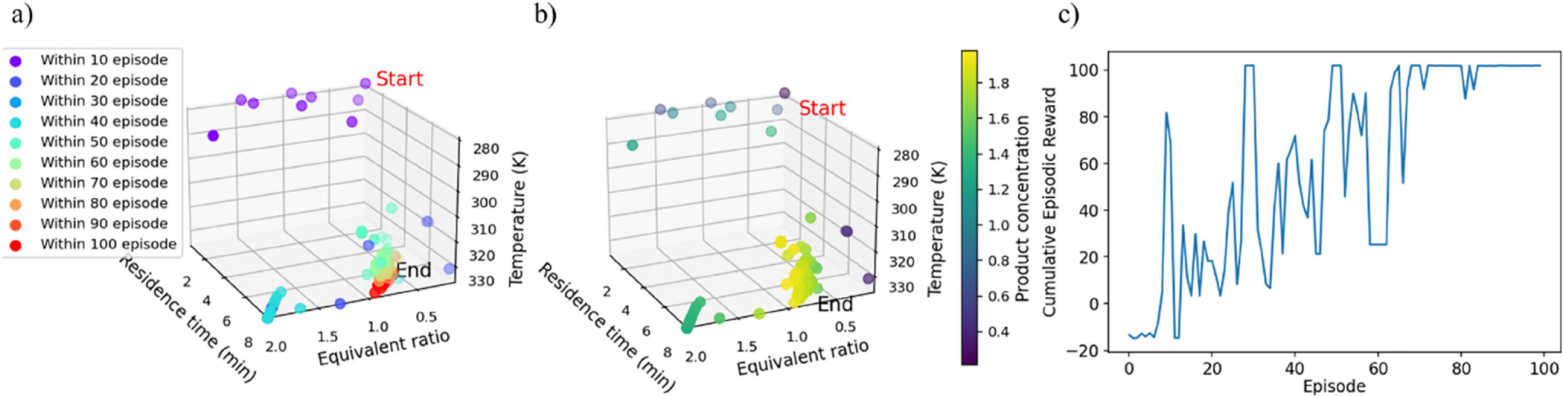
Training performance of the DDPG agent at adaptive noise.

In the case of learning rate adjustment, the dynamic adjustment of the learning rate ensures optimal progression throughout training. A higher effective learning rate at the start accelerates policy updates, allowing the agent to explore a broader range of state-action pairs and adapt to diverse operating conditions. As training progresses, the gradual reduction of the learning rate facilitates finer adjustments to the policy, improving stability and convergence. When combined with adaptive noise, this strategy improves performance by encouraging more efficient exploration and exploitation during the training phase. The action taken in the decision space and training performance are presented in Figure 14a, b, and c respectively. As shown in Figure 14a,b, the agent explores a broad range of conditions early in training, with data points scattered across the decision space within the first 50 episodes, especially at the start of the training process. After 50 episodes, there is a clear convergence toward the optimal conditions, represented by a cluster of data points toward the “End” region of the plots, indicating a shift towards exploitation. This transition aligns with the increasing cumulative episodic reward observed in Figure 14c, where a rapid increase occurs between episodes 20 and 50, followed by stabilization, and then converging toward a maximum between episodes 50 and 80. With the proposed dynamic hyperparameters tuning, the agent identifies the optimal residence time (min), equivalent ratio and temperature (K) at 3.8 min, 1.1 and 321 K, respectively. The adaptive hyperparameter tuning delivers a more effective balance between exploration and exploitation, resulting in best training performance and an optimal solution to the flow chemistry self-optimization problem. It becomes clear that the adaptive hyperparameter tuning leads to smoother learning trajectories, faster convergence, and overall performance improvements.

**Figure 14:**
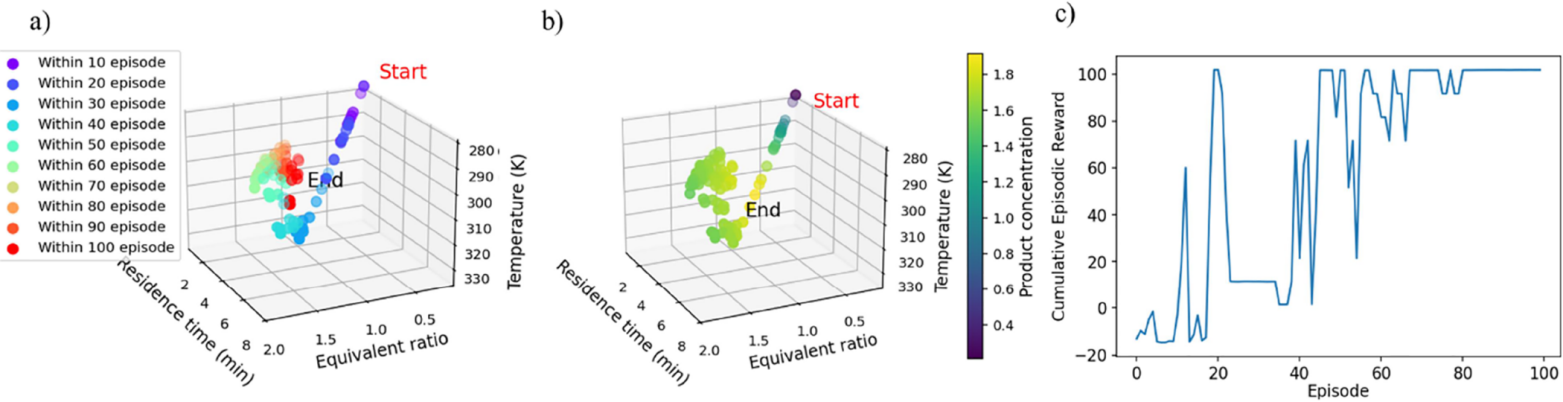
Training performance of the DDPG agent at adaptive learning rate.

### 3.5 Comparison of the DRL Agent against Gradient-Free Optimizers

The performance of the proposed RL strategy is compared against two widely used gradient-free optimization strategies (Nelder–Mead and SnobFit), for the self-optimization of the chemical reactions in flow. The exploration performance within decision space of these techniques is shown in Figure 15. The red stars in Figure 15a–c represent the optimal reaction conditions achieved by each optimizer. The figures show that each optimizer converges to optimal reaction conditions that deliver maximum product concentration of imine. It becomes clear that the proposed optimizer (RL based DDPG) outperforms Nelder–Mead and SnobFit based on the key performance indicators summarized in Table 6. This superiority is attributed to the intrinsic ability of DRL frameworks to manage the exploration-exploitation trade-off via policy gradient updates and deterministic action selection, a feature that gradient-free optimizers do not explicitly possess. Nelder–Mead identifies local optima through Simplex based refinement making it highly sensitive to initial guesses or starting conditions and therefore limited in its ability to explore the broader solution space. Conversely, SnobFit algorithms employs branch-and-fit strategies to achieve global optimization solution; however, this often comes at the cost of requiring a greater number of experimental evaluations.

**Figure 15:**
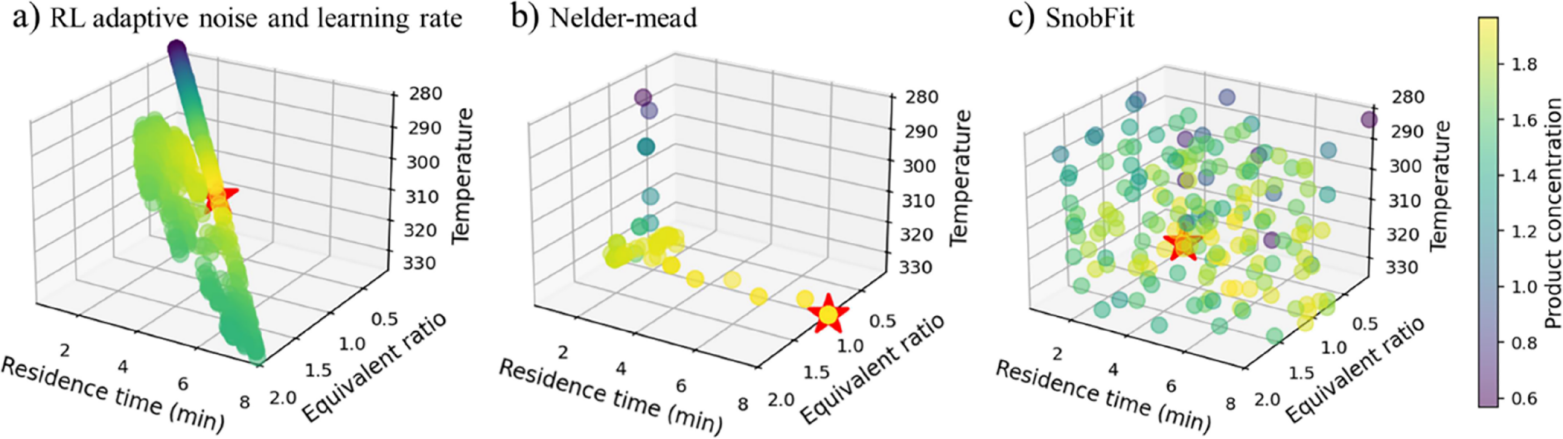
Performance of the DDPG agent vs Nelder–Mead and SnobFit.

**Table 6:** Comparison of the Key Performance Indicators of the Different Self-Optimization Methods^a^

			optimal solutions			
method	initial guess	number of experiments/iterations	manipulated variables	*c*_3_ (M)	experimental *c*_3_ (M)	computation cost (to achieve optimal solution)
Nelder-Mead	**Case 1**: ER_1/2_: 0.714, Res_*t*_: 0.66, *T*: 280.55	118	ER_1/2_: 1, Res_*t*_: 8, *T*: 330	1.81	1.79	∼66 s
	**Case 2**: ER_1/2_: 7.9, Res_t_: 1.48, *T*: 313.27	96	ER_1/2_: 0.63, Res_*t*_: 7, *T*: 330	1.65	1.50	∼54 s
SnobFit		250	ER_1/2_: 1, Res_*t*_: 6.8, *T*: 325	1.80	1.81	∼132 s
DRL (Bayesian)		85	ER_1/2_: 1.12, Res_*t*_: 2.1, *T*: 330.59	1.82	1.80	∼400 s
DRL (adaptive noise)		75	ER_1/2_: 1.12, Res_*t*_: 3.8, *T*: 320.59	1.83	1.81	∼400 s
DRL (adaptive noise and learning rate)		75	ER_1/2_: 1.1, Res_*t*_: 3.8, *T*: 321.57	1.84	1.83	∼400 s

a ER_1/2_: equivalent ratio, Res_*t*_: residence time, *T*: temperature, and *c*_3_: product concentration.

These results underscore the differences in efficiency, sensitivity to initial conditions, and the number of experiments required by each method to converge to optimal solutions. The proposed RL based approach converges to an optimal solution in a mere 60 experiments, a very small number when compared to Nelder–Mead and SnobFit, which require 120 and 250 experiments, respectively. However, Nelder–Mead algorithms identify optimal reaction conditions at the boundaries of temperature and residence time (residence time = 8 min, molar equivalent ratio = 1, and temperature = 330 K). When the initial guesses are altered, a different local solution is obtained (residence time = 7 min, molar equivalent ratio = 0.63, and temperature = 330 K). SnobFit, on the other hand, requires a higher number of experiments/iterations to fully explore the decision space and find the global solution as shown in Figure 15c.

The optimal conditions identified by the DRL-based agents, based on several hypermeter tuning methods, as well as SnobFit, and Nelder–Mead algorithms were experimentally implemented. The resulting experimental product concentrations are shown against predicted optimal values in Table 6. Overall, the experimental results are consistent with the optimal solutions identified by the model-based self-optimization approaches. As evidenced by the experimental results of the products concentrations, the DRL agents with the proposed hypermeter tuning methods delivered enhanced productivity compared to the standard methods. This consolidates the idea that the DRL agents, with the proposed tuning methods, not only reduce the required experiments but most importantly tend to converge to the global optimal solutions. Interestingly, the best self-optimization performance, showing specifically reduced experimentation costs and increased productivity, was obtained by the DRL agent under the proposed adaptive hypermeter tuning of two parameters, namely the noise and learning rate. These results highlight the great benefits of the new adaptive hyperparameter tuning in the general context of reinforcement learning and more specifically in the self-optimization of flow chemistry systems.

Regarding computation costs, the DRL methods with adaptive noise and learning rate tuning were more computationally intensive compared to traditional optimization methods like Nelder–Mead and SnobFit. Specifically, while the Nelder–Mead method showed quick convergence with a computation time of approximately 54–66 s (for 118–96 iterations), and SnobFit required around 132 s for 250 iterations, the DRL agents with adaptive tuning needed 250–400 s to achieve optimal solutions. Despite the increased computation cost, the DRL agents’ ability to dynamically balance exploration and exploitation allow them to reliably converge to global optima and significantly reduce the number of required experiments makes them highly beneficial in complex systems like flow chemistry. Therefore, while the computational cost is higher, the benefits of reduced experimentation, increased accuracy, and the ability to optimize intricate systems far outweigh these costs, reinforcing the value of DRL-based methods for self-optimization tasks. Details of this can be found in Section C of the Supporting Information (SI).

## 4 Conclusions

This paper proposed a new self-optimization strategy of a key flow chemistry process based on deep reinforcement learning. While the approach contributes to the development of the next generation plug-and-play technologies, it opens new opportunities for bias free and cost-effective process development and optimization strategies.

A state-of-the-art deep deterministic policy gradient (DDPG) agent was designed and trained to identify the optimal operating conditions that maximize the product concentration of the imine synthesis. The Bayesian optimization-based hyperparameter tuning demonstrated enhanced training performance compared to the traditional trial-and-error, which was evaluated based on the speed of convergence and training smoothness. These training key features were further enhanced using the proposed new adaptive dynamic hyperparameter tuning. To evaluate the effectiveness of different action policies, the DDPG agent was tested using two approaches: the noisy action policy and the ε-greedy action policy. Both policies demonstrated excellent performance in terms of computational speed and efficiency. However, the noisy action policy outperformed the ε-greedy approach by achieving a better balance between exploration and exploitation, as well as faster convergence to optimal solutions. This suggests that the DDPG agent equipped with the noisy action policy is more effective for this reaction optimization task.

Finally, the performance of the different DDPG agents, obtained based on the proposed architecture and hyperparameter tuning strategies, was validated and compared against state-of-the-art self-optimization methods, namely Nelder–Mead and SnobFit. Clearly, the DDPG agents demonstrated superior capabilities and exhibited enhanced tracking of the global optimal solution. The best performance was shown by the agent based the adaptive hyperparameter tuning which converged to the optimal solution in 60 experiments compared to 250 and up to 118 experiments in the case of SnobFit and Neder-Mead, respectively. Additionally, the experimental validations of the optimal profiles were conclusive and proved consistent with predicted model-based approaches. These key performance indicators underscore the capabilities of the proposed new DRL self-optimization strategies in the minimization of the experimental costs and associated environmental burdens.

The mathematical model identified based on the new experimental data was used as a training environmental to further reduce the experimentation costs. However, the approach can be fully implemented in real time, based solely on the experimentation or on a hybrid approach, which may be demonstrated in a future work. Moreover, the DRL can also be adapted to the multiobjective self-optimization case to help identify the set of the best compromises between different competing criteria.

## Supplementary Material



## Notes

Python packages used for reinforcement learning simulation, experimental data for parameter estimation, method used for parameter estimation, and optimization data for DRL (Bayesian, adaptive noise and adaptive noise and learning rate) (PDF)

## Nomenclature

*A*_*t*_: adaptation metric
*a*_*t*_: actions of the step, *t*
*C*_s*,j*_: concentration of the stock solution of reactant, *j*
*C*_*j*f_: concentration of each reactant in feed stream reactor, *j*
*C*_*p*_: average specific heat capacity
*C*_*p,i*_: specific heat capacity of *i* ^th^ species
*c*_3_: Product concentration
*dt*: time difference for noise
*E*_a_: activation energy
Ep: current episode
ER_1/2_: equivalent ratio
*E*_θ_: expected value based on the policy
*G*_*t*_: cumulative rewards
HP: hyperparameter
HP_min_: hyperparameter minimum value
HP_max_: hyperparameter maximum value
Δ*H*_react_: reaction enthalpy
*j*: reactant species
*k*: reaction rate constant
*k*_ref_: reference reaction rate coefficient
*L*(*Q* ^θ^): loss function critic network
*N*_total_: total number of steps
*N*_*t*_: training iteration
*Q*: total flow rate
*Q*_*j*_: individual reactant flow rate, *j*
*R*: universal gas constant
Res_*t*_: residence time
*r*: reaction rate
*r*_*t*_: instantaneous reward at the step, *t*
*s*_*t*_: state of the step, *t*
*T*: temperature inside tubular reactor
*T*_c_: coolant temperature
*T*_f_: feeding temperature
*T*_0_: initial reactor temperature
*T*_ref_: reference temperature
*t*: total reaction time
UA: overall heat transfer coefficient
*V*: volume of reactor
*v*_*z*_: total velocity of reactant flow in *z*-direction
*x*_*i*_: mole fraction of *i* ^th^ species
*z*: flow reaction
**Greek Letter**
α: decay rate
α_actor_: actor learning rate
α_critic_: critic learning rate
χ_at_: current value of noise
ε: greedy action
ε_min_: lowest probability of choosing a random action
γ: future reward discount factor
λ: exponential decay rate
μ: long-term mean
N: exploration noise
∇_θ^μ^_: actor network gradient
ρ: reaction mixture density
σ: volatility of noise/exploration noise parameter
τ: smoothing factor
θ: noise return rate
θ^μ^: actor network parameters
θ^Q^: critic network parameters
θ^μ’^: target actor network parameters
θ^Q’^: target critic network parameters
**Glossary**
DDPG: deep deterministic policy gradient
DNNs: deep neutral networks
DRL: deep reinforcement learning
HP: hyperparameter
HPO: hyperparameter optimization
LSTM: long–short-term memory
MINLP: mixed integer nonlinear programming
ML: machine learning
ODEs: ordinary differential equations
OVAT: one variable at a time
OU: Ornstein–Uhlenbeck
RL: reinforcement learning
SNOBFIT: stable noisy optimization by branch and fit
TD: temporal difference
TS-EMO: Thomson sampling efficient multi-objective
