# Supplementary material for: Deep Reinforcement Learning-Based Self-Optimization of Flow Chemistry
Source: ACS Eng Au. 2025 May 13;5(3):247–66. doi: 10.1021/acsengineeringau.5c00004 (PMC12183679; doi:10.1021/acsengineeringau.5c00004)
Supplement: Supplementary file 1 [file eg5c00004_si_002.pdf]

Supplementary information for

Deep Reinforcement Learning-Based Self-Optimization of Flow chemistry

Ashish Yewale[a], Yihui Yang[b], Neda Nazemifard[b], Charles D. Papageorgiou[b], Chris D. Rielly[a], Brahim Benyahia[a]*

[a] *Department of Chemical Engineering, Loughborough University, Loughborough, Leicestershire, LE11 3TU, United Kingdom*

[b] *Synthetic Molecule Process Development, Process Engineering and Technology, Takeda Pharmaceuticals International Company, 40 Landsdowne Street, Cambridge, Massachusetts 02139, United States*

b.benyahia@lboro.ac.uk

A.1 Python packages used for RL simulation

- *TensorFlow (version 2.18.0) was used for the deep reinforcement learning framework.*
- *NumPy (version 1.26.0) was employed for data manipulation.*
- *SciPy (version 1.12.0) was used for numerical computations.*
- *gym (version 0.26.2) was utilized for the reinforcement learning environment.*
- *matplotlib (version 3.9.2) was used for data visualization.*
- *pandas (version 2.2.3) was employed for data processing.*
- *json (version 4.23) was used for data storage.*
- *Optuna (version 4.2.1) was used for hyperparameter optimization.*

B.1 Experimental data for parameter estimation

**Temperature 288 K**			**Temperature 298 K**			**Temperature 308 K**			**Temperature 318 K**		
Time	Reactant	Product	Time	Reactant	Product	Time	Reactant	Product	Time	Reactant	Product
0.5	1.9	0.1	0.5	1.836	0.164	0.5	1.728	0.272	0.5	1.71	0.29
1	1.621	0.379	1	1.523	0.477	1	1.378	0.622	1	1.19	0.81
1.5	1.251	0.749	1.5	1.207	0.793	1.5	1.051	0.949	1.5	0.795	1.205

2	1.049	0.951	2	0.963	1.037	2	0.792	1.208	2	0.612	1.388
2.5	0.909	1.091	2.5	0.787	1.213	2.5	0.642	1.358	2.5	0.499	1.501
3	0.779	1.221	3	0.645	1.355	3	0.537	1.463	3	0.427	1.573
3.5	0.698	1.302	3.5	0.575	1.425	3.5	0.468	1.532	3.5	0.379	1.621
4	0.628	1.372	4	0.509	1.491	4	0.421	1.579	4	0.332	1.668
4.5	0.575	1.425	4.5	0.458	1.542	4.5	0.375	1.625	4.5	0.296	1.704
5	0.518	1.482	5	0.418	1.582	5	0.339	1.661	5	0.268	1.732
5.5	0.482	1.518	5.5	0.381	1.619	5.5	0.303	1.697	5.5	0.243	1.757
6	0.454	1.546	6	0.353	1.647	6	0.278	1.722	6	0.227	1.773
6.5	0.421	1.579	6.5	0.329	1.671	6.5	0.257	1.743	6.5	0.203	1.797
7	0.395	1.605	7	0.301	1.699	7	0.239	1.761	7	0.185	1.815
7.5	0.376	1.624	7.5	0.285	1.715	7.5	0.221	1.779	7.5	0.176	1.824
8	0.358	1.642	8	0.268	1.732	8	0.209	1.791	8	0.163	1.837
8.5	0.339	1.661	8.5	0.251	1.749	8.5	0.198	1.802	8.5	0.151	1.849
9	0.322	1.678	9	0.243	1.757	9	0.19	1.81	9	0.144	1.856
9.5	0.305	1.695	9.5	0.228	1.772	9.5	0.181	1.819	9.5	0.136	1.864
10	0.288	1.712	10	0.218	1.782	10	0.168	1.832	10	0.127	1.873
10.5	0.276	1.724	10.5	0.209	1.791	10.5	0.162	1.838	10.5	0.119	1.881
11	0.266	1.734	11	0.199	1.801	11	0.153	1.847	11	0.115	1.885
11.5	0.258	1.742	11.5	0.191	1.809	11.5	0.147	1.853	11.5	0.109	1.891
12	0.248	1.752	12	0.185	1.815	12	0.141	1.859	12	0.105	1.895
12.5	0.236	1.764	12.5	0.178	1.822	12.5	0.138	1.862	12.5	0.101	1.899
13	0.227	1.773	13	0.171	1.829	13	0.133	1.867	13	0.097	1.903
13.5	0.218	1.782	13.5	0.167	1.833	13.5	0.128	1.872	13.5	0.094	1.906
14	0.209	1.791	14	0.161	1.839	14	0.1235	1.8765	14	0.092	1.908
14.5	0.201	1.799	14.5	0.158	1.842	14.5	0.121	1.879	14.5	0.087	1.913
15	0.195	1.805	15	0.151	1.849	15	0.118	1.882	15	0.083	1.917

15.5	0.19	1.81	15.5	0.147	1.853	15.5	0.115	1.885	15.5	0.079	1.921
16	0.182	1.818	16	0.141	1.859	16	0.111	1.889	16	0.077	1.923
16.5	0.179	1.821	16.5	0.138	1.862	16.5	0.109	1.891	16.5	0.075	1.925
17	0.175	1.825	17	0.134	1.866	17	0.105	1.895	17	0.073	1.927
17.5	0.171	1.829	17.5	0.129	1.871	17.5	0.102	1.898	17.5	0.071	1.929
18	0.166	1.834	18	0.126	1.874	18	0.098	1.902	18	0.069	1.931
18.5	0.161	1.839	18.5	0.123	1.877	18.5	0.094	1.906	18.5	0.067	1.933
19	0.155	1.845	19	0.117	1.883	19	0.091	1.909	19	0.065	1.935
19.5	0.15	1.85	19.5	0.111	1.889	19.5	0.089	1.911	19.5	0.064	1.936
20	0.145	1.855	20	0.107	1.893	20	0.087	1.913	20	0.063	1.937
20.5	0.147	1.853	20.5	0.104	1.896	20.5	0.085	1.915	20.5	0.062	1.938
21	0.143	1.857	21	0.099	1.901	21	0.083	1.917	21	0.061	1.939
21.5	0.139	1.861	21.5	0.098	1.902	21.5	0.081	1.919	21.5	0.0605	1.9395
22	0.135	1.865	22	0.096	1.904	22	0.08	1.92	22	0.059	1.941
22.5	0.131	1.869	22.5	0.095	1.905	22.5	0.079	1.921	22.5	0.058	1.942
23	0.128	1.872	23	0.092	1.908	23	0.078	1.922	23	0.057	1.943
23.5	0.128	1.872	23.5	0.09	1.91	23.5	0.077	1.923	23.5	0.056	1.944
24	0.123	1.877	24	0.087	1.913	24	0.076	1.924	24	0.055	1.945
24.5	0.121	1.879	24.5	0.086	1.914	24.5	0.075	1.925	24.5	0.054	1.946
25	0.119	1.881	25	0.084	1.916	25	0.074	1.926	25	0.0545	1.9455
25.5	0.117	1.883	25.5	0.078	1.922	25.5	0.073	1.927	25.5	0.054	1.946
26	0.115	1.885	26	0.076	1.924	26	0.072	1.928	26	0.0538	1.9462
26.5	0.113	1.887	26.5	0.075	1.925	26.5	0.071	1.929	26.5	0.0535	1.9465
27	0.111	1.889	27	0.074	1.926	27	0.07	1.93	27	0.053	1.947
27.5	0.109	1.891	27.5	0.073	1.927	27.5	0.069	1.931	27.5	0.0525	1.9475
28	0.107	1.893	28	0.072	1.928	28	0.067	1.933	28	0.052	1.948
28.5	0.105	1.895	28.5	0.07	1.93	28.5	0.065	1.935	28.5	0.0518	1.9482

29	0.103	1.897	29	0.069	1.931	29	0.063	1.937	29	0.0515	1.9485
29.5	0.101	1.899	29.5	0.068	1.932	29.5	0.061	1.939	29.5	0.0513	1.9487
30	0.096	1.904	30	0.067	1.933	30	0.058	1.942	30	0.05	1.95

B.2 Method used for parameter estimation

As per the methodology used, the parameter estimation was conducted using the available experimental data. Specifically, the maximum likelihood function, assuming Gaussian noise in the measurements, was employed to estimate the parameters. The objective function used for parameter estimation is given by:

$$J(\boldsymbol{\theta}) = \sum_{i=1}^{n_y} \sum_{j=1}^{n_t} \frac{[y_i(t_j) - h_i(\boldsymbol{x}(t_j), \boldsymbol{\theta})]^2}{\sigma_i^2}. \quad (1)$$

The best parameter estimates were obtained by minimising the objective function $J(\boldsymbol{\theta})$:

$$\hat{\boldsymbol{\theta}} = argmin[J(\boldsymbol{\theta})] \quad (2)$$

where:

- $\theta=(\theta_1, \ldots, \theta_{n\theta})$ is the vector of unknown model parameters,
- y_i is the vector of the ith measured output of the system (observables),
- x represents the vector of state variables,
- t_j is the jth sampling time,
- n_x, n_u, n_y, n_θ are the number of state variables, input variables, output variables, and parameters, respectively. "

C.1 Optimization data for DRL (bayesian)

Residence time	Equivalent ratio	Temperature	Concentration
0.50	0.10	278.00	0.21
0.54	0.10	278.69	0.23
0.80	0.13	282.89	0.36
1.21	0.24	291.77	0.72
1.09	0.41	307.03	1.10
0.36	0.50	319.16	1.21
0.50	0.82	332.22	1.59
0.43	1.46	364.72	1.62
0.69	1.96	392.09	1.35
0.57	2.00	393.00	1.33
0.45	2.00	393.00	1.33
0.51	2.00	392.96	1.33

0.62	2.00	392.81	1.33
0.51	1.99	392.51	1.34
0.64	2.00	393.00	1.33
0.48	2.00	393.00	1.33
0.54	2.00	393.00	1.33
0.55	2.00	392.98	1.33
0.43	2.00	393.00	1.33
0.39	1.99	392.42	1.34
0.51	2.00	392.93	1.33
0.41	1.98	391.63	1.34
0.42	1.98	391.79	1.34
0.49	2.00	393.00	1.33
0.43	2.00	393.00	1.33
0.57	2.00	393.00	1.33
0.45	1.99	392.12	1.34
0.67	1.96	391.02	1.35
0.64	1.54	390.07	1.57
0.47	1.37	376.80	1.68
0.41	1.37	364.36	1.68
0.60	1.30	359.11	1.71
0.52	1.32	354.85	1.69
0.44	1.29	353.70	1.70
0.35	1.36	359.63	1.68
0.59	1.30	357.75	1.71
0.79	1.35	360.92	1.68
0.48	1.40	365.96	1.66
0.57	1.49	371.90	1.60
0.48	1.57	376.15	1.56
0.45	1.46	369.41	1.62
0.45	1.34	359.26	1.68
0.42	1.15	344.84	1.71
0.64	1.00	345.81	1.74
0.51	0.86	346.58	1.72
0.43	0.91	352.81	1.77
0.62	0.90	358.53	1.80
0.56	0.97	362.59	1.84
0.59	1.07	365.46	1.84
0.45	1.14	363.46	1.81
0.53	1.19	364.83	1.79
0.55	1.28	368.31	1.74
0.43	1.28	368.09	1.74
0.55	1.37	375.48	1.68
0.42	1.38	376.21	1.68
0.49	1.44	375.36	1.64

0.59	1.53	380.73	1.58
0.49	1.39	372.64	1.67
0.56	1.40	375.51	1.67
0.60	1.38	374.70	1.68
0.33	1.31	373.47	1.73
0.54	1.34	374.71	1.70
0.70	1.27	372.87	1.75
0.42	1.32	374.95	1.72
0.32	1.24	373.89	1.77
0.59	1.25	372.61	1.76
0.24	1.25	374.60	1.76
0.65	1.28	379.14	1.75
0.28	1.15	372.65	1.83
0.48	1.23	376.04	1.78
0.32	1.23	376.97	1.78
0.33	1.22	374.01	1.79
0.70	1.26	377.27	1.76
0.37	1.21	373.79	1.79
0.60	1.23	372.71	1.78
0.55	1.23	375.52	1.79
0.57	1.15	372.91	1.83
0.63	1.27	379.16	1.75
0.60	1.17	375.44	1.82
0.48	1.15	371.11	1.83
0.61	1.19	377.58	1.81
0.57	1.19	372.67	1.80
0.47	1.23	371.09	1.78
0.41	1.16	372.89	1.82
0.39	1.15	377.17	1.84
0.46	1.16	374.69	1.83
0.47	1.21	375.41	1.79
0.20	1.16	373.38	1.83
0.46	1.10	363.54	1.82
0.53	1.17	365.37	1.80
0.50	1.14	361.50	1.80
0.38	1.16	364.65	1.80
0.52	1.10	361.02	1.82
0.49	1.12	362.35	1.81
0.63	1.08	359.14	1.81
0.59	1.06	354.43	1.79
0.45	1.06	358.83	1.82
0.58	1.07	359.16	1.82
0.37	1.10	359.11	1.81
0.44	1.13	357.47	1.79

C.2 Optimization data for DRL (adaptive noise)

Residence time	Equivalent ratio	Temperature	Concentration
0.50	0.10	278.00	0.21
0.50	0.10	278.00	0.21
0.80	0.34	278.00	0.64
0.63	0.82	278.00	0.74
1.75	2.00	278.00	1.03
0.50	1.38	278.00	0.64
0.50	0.78	278.00	0.64
2.11	0.95	278.00	1.26
2.46	0.43	278.00	1.05
7.42	1.15	372.67	1.86
6.26	0.35	364.05	1.04
5.58	0.10	355.25	0.36
7.23	0.10	381.09	0.36
7.57	1.28	386.35	1.76
8.00	2.00	393.00	1.33
8.00	2.00	393.00	1.33
7.87	1.97	391.02	1.35
8.00	2.00	393.00	1.33
8.00	2.00	393.00	1.33
7.69	1.92	388.23	1.37
7.94	1.99	392.11	1.34
8.00	2.00	393.00	1.33
7.83	1.96	390.44	1.35
7.88	1.97	391.19	1.35
8.00	2.00	393.00	1.33
8.00	2.00	393.00	1.33
8.00	2.00	393.00	1.33
7.95	1.99	392.22	1.34
7.92	1.98	391.77	1.34
8.00	2.00	393.00	1.33
8.00	2.00	393.00	1.33
8.00	2.00	392.98	1.33
7.73	1.93	388.83	1.36
7.62	1.90	387.12	1.38
7.21	1.80	380.87	1.43
7.85	1.96	390.71	1.35
7.31	1.82	382.47	1.42
7.68	1.87	388.03	1.39
8.00	0.80	391.15	1.78
7.84	0.93	348.23	1.93
7.98	1.20	349.77	1.82
8.00	1.19	344.73	1.83

8.00	1.03	351.49	1.96
7.61	0.91	356.57	1.91
7.62	0.84	357.73	1.82
8.00	0.95	370.23	1.94
7.80	0.83	363.39	1.81
8.00	0.94	371.18	1.94
8.00	0.99	373.12	1.99
8.00	0.90	364.59	1.90
7.94	0.86	364.10	1.85
8.00	0.98	374.54	1.98
8.00	0.93	368.02	1.93
8.00	0.92	368.13	1.92
7.94	0.89	367.23	1.89
7.86	0.85	363.99	1.84
7.67	0.74	362.75	1.70
7.71	0.79	363.10	1.76
7.93	0.81	366.06	1.79
8.00	0.85	374.93	1.84
8.00	0.82	373.04	1.80
8.00	0.83	376.21	1.81
7.89	0.78	372.29	1.75
8.00	0.86	377.70	1.84
8.00	0.86	369.03	1.84
7.75	0.70	369.60	1.64
7.87	0.77	376.19	1.74
7.91	0.89	369.21	1.88
8.00	0.90	376.04	1.89
7.99	0.83	372.96	1.82
8.00	0.87	371.77	1.86
8.00	0.94	380.38	1.94
8.00	0.91	381.69	1.90
7.72	0.77	372.29	1.74
8.00	0.81	379.67	1.79
7.77	0.80	375.73	1.78
7.56	0.71	369.37	1.66
8.00	0.88	381.49	1.87
8.00	0.81	378.82	1.79
7.98	0.83	378.55	1.82
7.47	0.68	368.43	1.62
8.00	0.81	380.03	1.79
7.82	0.77	374.42	1.74
7.65	0.76	372.59	1.72
7.88	0.81	375.45	1.79
7.88	0.74	378.10	1.71

8.00	0.77	378.31	1.75
8.00	0.81	381.41	1.79
7.79	0.72	376.94	1.67
8.00	0.91	385.02	1.91
8.00	0.86	381.79	1.85
7.87	0.76	376.14	1.73
8.00	0.82	381.59	1.81
7.87	0.78	376.93	1.75
8.00	0.87	385.50	1.86
8.00	0.85	385.36	1.84
8.00	0.81	382.82	1.79
8.00	0.85	385.75	1.84
8.00	0.95	390.14	1.95
8.00	0.84	384.60	1.83

C.3 Optimization data for DRL (adaptive noise and learning rate)

Residence time	Equivalent ratio	Temperature	Concentration
0.50	0.10	278.00	0.21
2.70	0.66	311.58	1.56
2.42	0.58	307.24	1.45
2.00	0.48	300.85	1.25
2.40	0.58	307.06	1.44
0.50	0.10	278.00	0.21
0.50	0.10	278.00	0.21
0.90	0.20	284.17	0.54
1.22	0.28	288.91	0.78
1.18	0.27	288.21	0.75
1.95	0.46	299.86	1.22
3.12	0.75	317.61	1.70
1.55	0.35	293.39	0.98
0.50	0.10	278.00	0.21
1.98	0.46	299.78	1.22
1.39	0.31	290.42	0.87
1.54	0.35	293.09	0.98
1.53	0.35	292.92	0.97
3.75	0.90	326.03	1.86
3.34	0.80	319.81	1.75
5.07	1.23	346.11	1.79
6.21	1.53	363.79	1.58
7.09	1.76	377.82	1.45
7.74	1.93	388.05	1.37
7.73	1.93	387.92	1.37
7.69	1.93	387.48	1.37

7.37	1.86	382.49	1.40
7.15	1.81	379.11	1.42
7.16	1.83	379.16	1.41
6.86	1.77	374.55	1.44
7.07	1.84	377.82	1.41
6.75	1.79	372.93	1.44
7.08	1.89	377.95	1.38
6.72	1.83	372.50	1.41
7.08	1.95	378.03	1.36
6.82	1.92	374.06	1.37
6.91	1.97	375.56	1.35
5.63	1.72	355.98	1.47
5.64	1.76	356.27	1.45
4.05	1.42	332.00	1.65
4.86	1.67	344.63	1.50
3.89	1.46	329.93	1.62
3.99	1.53	331.57	1.58
4.38	1.67	337.52	1.50
3.31	1.41	321.23	1.65
2.56	1.21	309.83	1.70
2.91	1.33	315.21	1.68
3.00	1.39	316.66	1.65
3.12	1.44	318.41	1.63
1.98	1.11	301.05	1.60
2.39	1.24	307.29	1.66
3.03	1.43	317.03	1.63
3.23	1.52	320.11	1.58
3.02	1.46	316.92	1.61
2.97	1.45	316.05	1.62
2.23	1.23	304.68	1.63
2.33	1.24	306.04	1.65
2.95	1.45	315.47	1.62
2.67	1.35	311.04	1.65
2.62	1.33	310.21	1.66
2.19	1.19	303.52	1.63
2.19	1.17	303.48	1.64
2.27	1.20	304.62	1.64
2.54	1.28	308.47	1.66
2.62	1.30	309.46	1.66
2.14	1.15	301.83	1.62
2.44	1.24	305.95	1.66
2.57	1.27	307.76	1.67
2.39	1.20	304.63	1.66
2.82	1.33	310.82	1.67

2.54	1.23	306.38	1.67
2.50	1.21	305.56	1.67
2.59	1.23	306.65	1.67
2.33	1.14	302.78	1.65
2.48	1.17	304.81	1.67
2.09	1.05	298.73	1.59
2.64	1.19	307.03	1.70
2.59	1.15	306.11	1.70
2.01	0.99	297.20	1.56
2.42	1.08	303.29	1.67
2.35	1.04	302.27	1.66
2.76	1.15	308.30	1.73
2.34	1.02	302.13	1.66
2.85	1.15	309.53	1.74
3.01	1.17	312.05	1.75
2.75	1.08	308.01	1.74
3.07	1.15	312.85	1.77
3.21	1.18	315.00	1.77
3.01	1.11	311.81	1.78
2.91	1.07	310.28	1.77
3.05	1.09	312.33	1.79
2.70	0.97	307.05	1.74
2.79	0.98	308.41	1.76
3.10	1.05	313.06	1.80
3.06	1.02	312.19	1.80
3.07	1.01	312.42	1.80
3.40	1.08	317.39	1.83
4.07	1.23	327.18	1.78
4.13	1.23	327.93	1.79
3.72	1.10	321.58	1.85
